# Optimal Perioperative Nutrition Therapy for Patients Undergoing Pancreaticoduodenectomy: A Systematic Review with a Component Network Meta-Analysis

**DOI:** 10.3390/nu13114049

**Published:** 2021-11-12

**Authors:** Shang-Yu Wang, Yu-Liang Hung, Chih-Chieh Hsu, Chia-Hsiang Hu, Ruo-Yi Huang, Chang-Mu Sung, Yan-Rong Li, Hao-Wei Kou, Ming-Yang Chen, Shih-Chun Chang, Chao-Wei Lee, Chun-Yi Tsai, Keng-Hao Liu, Jun-Te Hsu, Chun-Nan Yeh, Ta-Sen Yeh, Tsann-Long Hwang, Yi-Yin Jan, Miin-Fu Chen

**Affiliations:** 1 Division of General Surgery, Department of Surgery, Chang Gung Memorial Hospital, Taoyuan City 333, Taiwan; d0100106@cgu.edu.tw (S.-Y.W.); brianhung24241111@gmail.com (Y.-L.H.); m8283@adm.cgmh.org.tw (C.-C.H.); medicon36@gmail.com (C.-H.H.); mr1309@cgmh.org.tw (R.-Y.H.); b9602039@cgmh.org.tw (H.-W.K.); Bhreangel@gmail.com (M.-Y.C.); b9302071@cgmh.org.tw (S.-C.C.); alanlee@cgmh.org.tw (C.-W.L.); m7202@cgmh.org.tw (C.-Y.T.); kenghao@cgmh.org.tw (K.-H.L.); hsujt2813@cgmh.org.tw (J.-T.H.); tsy471027@cgmh.org.tw (T.-S.Y.); hwangtl@cgmh.org.tw (T.-L.H.); janyy@cgmh.org.tw (Y.-Y.J.); chenmf@cgmh.org.tw (M.-F.C.); 2 Graduate Institute of Clinical Medical Sciences, Chang Gung University, Taoyuan City 333, Taiwan; 3 Division of Gastroenterology and Hepatology, Department of Internal Medicine, Chang Gung Memorial Hospital, Taoyuan City 333, Taiwan; andrew3329@gmail.com; 4 Division of Endocrinology and Metabolism, Department of Internal Medicine, Chang Gung Memorial Hospital, Taoyuan City 333, Taiwan; malyr8252@gmail.com

**Keywords:** pancreaticoduodenectomy, network meta-analysis, nutrition therapy, postoperative pancreatic fistula, immunonutrition

## Abstract

Numerous strategies for perioperative nutrition therapy for patients undergoing pancreaticoduodenectomy (PD) have been proposed. This systematic review aimed to summarize the current relevant published randomized controlled trials (RCTs) evaluating different nutritional interventions via a traditional network meta-analysis (NMA) and component network meta-analysis (cNMA). EMBASE, MEDLINE, the Cochrane Library, and ClinicalTrials.gov were searched to identify the RCTs. The evaluated nutritional interventions comprised standard postoperative enteral nutrition by feeding tube (Postop-SEN), preoperative enteral feeding (Preop-EN), postoperative immunonutrients (Postop-IM), preoperative oral immunonutrient supplement (Preop-IM), and postoperative total parenteral nutrition (TPN). The primary outcomes were general, infectious, and noninfectious complications; postoperative pancreatic fistula (POPF); and delayed gastric emptying (DGE). The secondary outcomes were mortality and length of hospital stay (LOS). The NMA and cNMA were conducted with a frequentist approach. The results are presented as odds ratios (ORs) and 95% confidence intervals (CIs). Two primary outcomes, infectious complications and POPF, were positively influenced by nutritional interventions. Preop-EN plus Postop-SEN (OR 0.11; 95% CI 0.02~0.72), Preop-IM (OR 0.22; 95% CI 0.08~0.62), and Preop-IM plus Postop-IM (OR 0.11; 95% CI 0.03~0.37) were all demonstrated to be associated with a decrease in infectious complications. Postop-TPN (OR 0.37; 95% CI 0.19~0.71) and Preop-IM plus Postop-IM (OR 0.21; 95% CI 0.06~0.77) were clinically beneficial for the prevention of POPF. While enteral feeding and TPN may decrease infectious complications and POPF, respectively, Preop-IM plus Postop-IM may provide the best clinical benefit for patients undergoing PD, as this approach decreases the incidence of both the aforementioned adverse effects.

## 1. Introduction

Pancreaticoduodenectomy (PD) is one of the most extensive and radical surgical procedures for periampullary tumors. Patients are predisposed to a high nutritional risk due to the disruption and reconstruction of biliary, pancreatic, and upper gastrointestinal continuity, the complexity of the procedure, and metabolic derangement due to pancreatic resection [1]. In addition, patients may be prevented from obtaining nutrition orally or even via gastric tubes due to surgical complications. The incidence of PD-related complications has been reported to range from 20% to 30%, and the incidence of insufficiency of pancreaticojejunostomy, namely, postoperative pancreatic fistula (POPF), ranges from 14% to 20% [2]. Therefore, perioperative nutrition therapy has been recognized as critical for facilitating patient recovery from surgical stress, the management of surgical complications, and long-term patient outcomes after pancreatic surgery [3]. In addition to the meticulous assessment of nutritional risk, perioperative selection among various nutritional formulas and routes of nutrition administration are important considerations for clinicians. Currently available perioperative nutrition therapies include immunonutrition, total or partial parental nutrition, and oral or tube-based nutrition. In addition, the timing of delivery of nutrition therapy is critical, and the clinical condition of individual patients should be taken into consideration. However, there are conflicts between the different nutrition therapy strategies in the published studies to date [4,5,6,7]. In this review, we investigated whether any specific nutrition therapy is superior in terms of PD surgery complications. We focused on common surgical complications, and the most important and specific complication related to PD, postoperative pancreatic fistula (POPF). We systematically reviewed all the searchable evidence and used the traditional network meta-analysis (NMA) and component network meta-analysis (cNMA) techniques to conclude the best evidence from the eligible randomized control trials (RCTs).

## 2. Methods

### 2.1. Search Strategy and Selection Criteria

This systematic review with a network meta-analysis (SR-NMA) was conducted and reported according to the Preferred Reporting Items for Systematic Reviews and Meta-Analyses (PRISMA) NMA extension [8]. This study was registered in PROSPERO (CRD42021279285). Two authors searched all RCTs in EMBASE, MEDLINE, the Cochrane Library, and ClinicalTrials.gov (Y-L.H. and C-C.H.). The following relevant entry terms with Boolean logical combinations were applied to search the different databases: “pancreatic cancer”, “pancreaticoduodenectomy”, “diet therapy”, “enteric feeding”, “total parenteral nutrition”, “glutamine”, “arginine”, “nucleotides”, and “fatty acid”. The detailed search strategies with adequate filters are demonstrated in the Supplementary Materials. The articles were searched up to the final date of 20th July 2021. Duplications were autoremoved by Mendeley (Version 1.19.8). Two authors (Y.-L.H. and C.-C.H.) performed the initial article screening and review, and any discrepancy was resolved by consensus or consultation with another independent author (C.-N.Y.). Studies were included according to the following criteria: patients receiving PD or pylorus-preserving PD (PPPD) with nutrition therapy as an intervention for clinical outcome comparison. Studies were excluded according to the following criteria: (1) patients with metastatic or nonresectable cancers, gastric cancers, esophageal cancers, and other lower gastrointestinal cancers; (2) studies reporting outcomes from different published studies using the same dataset (only the study with the longest timeframe was included); (3) studies in which control group data were collected retrospectively; (4) studies for which full-text articles could not be retrieved; (5) studies with inadequate outcome data available for data synthesis; and (6) studies including an irrelevant comparison group. No restrictions were applied based on publication year, language, or patient age, sex, or race. The final enrolled articles for statistical synthesis were approved by another independent author (S.-Y.W.).

### 2.2. Outcome Measures

Our primary outcomes were the impact of nutrition therapy on perioperative complications, including general, infectious, and noninfectious complications, POPF, and delayed gastric emptying (DGE). POPF was mainly defined according to the International Study Group of Pancreatic Fistula (ISGPF) guidelines [9]. The secondary outcomes were mortality and length of hospital stay (LOS).

### 2.3. Data Extraction and Quality Assessment

Data extraction from enrolled RCTs included patient age and sex; the incidence of general, infectious, and noninfectious complications; the occurrence of POPF and DGE; the mortality rate; and LOS. All data presented as the medians and interquartile range were converted to the means and standard deviation [10].

Two independent authors performed the quality assessment with RevMan 5.4 (C.-Y.T. and S.-Y.W.). The risk of bias tool was used for the quality assessment using the following seven domains: (1) random sequence generation, (2) allocation concealment, (3) blinding of participants and personnel, (4) blinding of outcome assessment, (5) incomplete outcome data, (6) selective reporting, and (7) other bias [11]. All domains were judged as high risk, unclear risk, or low risk. Any disagreement was resolved by consensus or seeking consultation with another author (Y.-Y.J.).

### 2.4. Statistical Methods

We conducted NMA with a frequentist approach. We analyzed pooled odds ratios (ORs) for categorical data with 95% confidence intervals (CIs). For continuous data, we analyzed the mean difference (MD) with 95% CIs. Zero events were handled by adding 0.5 to each 2-by-2 table according to the Cochrane Handbook for Systematic Reviews of Interventions [12]. Many of the enrolled studies were designed to evaluate single nutrition therapy or combined nutrition therapy. While conducting the traditional NMA, the use of one nutrition therapy involving complex treatment with a combination of several interventions may be considered one intervention. Therefore, the potential effect of a single nutritional intervention may be underestimated. Component NMA (cNMA) models were applied to investigate this effect to ensure the precise evaluation of the additive effects of each nutritional intervention in our study [13]. Heterogeneity was assessed using I^2^ statistics, and I^2^ statistics greater than 50% indicated substantial heterogeneity. In general, fixed effects models (FEMs) were applied for evidence synthesis. Random effects models (REMs) were applied when substantial heterogeneity was present. Inconsistencies between direct and indirect evidence were examined by separating indirect from direct evidence (the SIDE approach) [14]. Funnel plots and Egger’s test were used to assess potential publication bias. The relative ranking probabilities for each nutrition therapy and the surface under the cumulative ranking (SUCRA) were calculated to determine the relative effect of each nutrition therapy. The certainty of the evidence was assessed by GRADE guidelines [15]. A result with a *p*-value below 0.05 was considered to be statistically significant. The statistical analyses were conducted with the “netmeta” package in R (Version 1.4.1717, the R Foundation for Statistical Computing, Vienna, Austria).

## 3. Results

### 3.1. Study Selection and Study Characteristics

**Figure 1:**
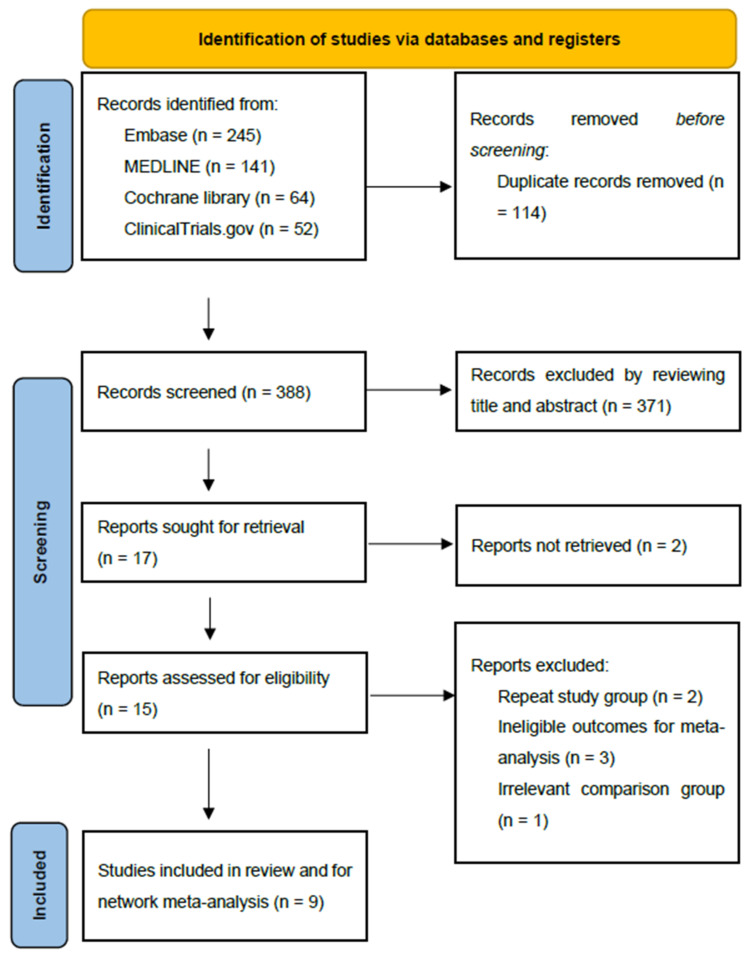
PRISMA 2020 flow diagram for NMA. PRISMA: Preferred Reporting Items for Systematic Reviews and Meta-Analyses; NMA: network meta-analysis.

We screened EMBASE, MEDLINE, the Cochrane Library, and ClinicalTrial.com and retrieved 502 records (Figure 1). After removing 114 duplicate records, the titles and abstracts were reviewed, and only 17 studies were retained. Further evaluation was performed, and only nine eligible RCTs were included for the subsequent analysis [6,16,17,18,19,20,21,22,23]. The nine relevant RCTs included were conducted in seven countries across Europe and the Asia-Pacific region between 2000 and 2019. In total, 724 patients were included. The nutrition therapies involved included standard postoperative enteral nutrition by feeding tube (Postop-SEN), preoperative enteral feeding (Preop-EN), postoperative immunonutrients (Postop-IM), preoperative oral immunonutrient supplement (Preop-IM), and postoperative total parenteral nutrition (TPN). The immunonutrient formula used in all the eligible studies was IMPACT^®^ (Nestlé Health Science, Avenue Nestlé 55, 1800 Vevey, Switzerland). The dosage of the nutrition therapies, such as calorie numbers, protein levels, and lipid formulas in the individual studies, was prescribed according to the needs of the individual patients and the availability of nutritional formulas at the individual institutions. Other details regarding all nine studies are summarized in Table 1.

### 3.2. Risk of Bias within Studies

According to the domains of bias defined by RoB 1.0, all the studies had blinding issues, both for the participants and outcome assessments. This issue might have resulted from the difficulty of blinding participants and investigators to the approach when prescribing nutrition therapy. In addition, one of the nine studies had potential randomization and allocation issues [22]. The results of the bias assessment are shown in Supplementary Figure S1.

### 3.3. Primary Outcomes

**Figure 2:**
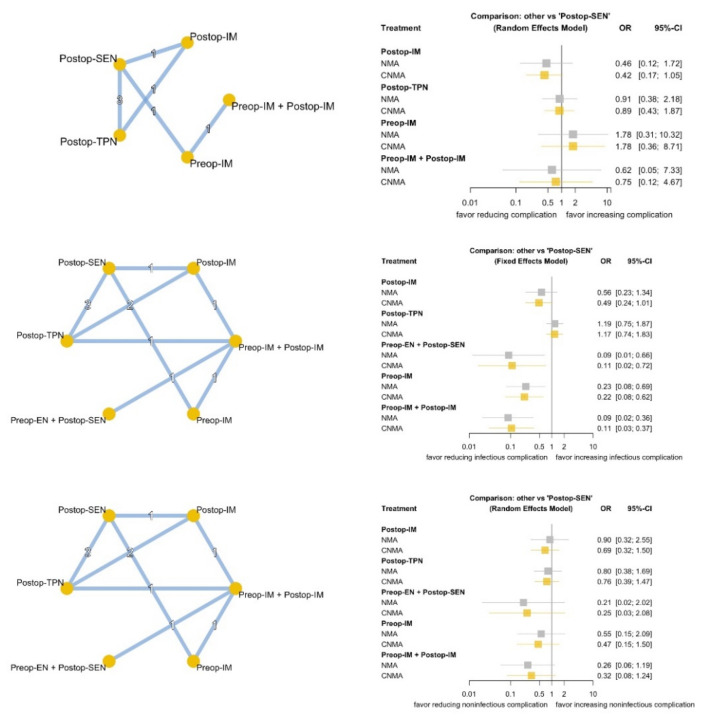
Network diagram and forest plot of NMA and cNMA for general, infectious, and noninfectious complications. NMA: network meta-analysis; cNMA: component network meta-analysis.

None of the nutrition therapies provided clinical benefit in terms of general complications (Figure 2). We further categorized complications into infectious and noninfectious complications. No individual nutrition therapy demonstrated a benefit in terms of noninfectious complications. Preop-EN plus Postop-SEN (NMA: OR 0.09, 95% CI 0.01~0.66; cNMA: OR 0.11, 95% CI 0.02~0.72), Preop-IM (NMA: OR 0.23, 95% CI 0.08~0.69; cNMA: OR 0.22, 95% CI 0.08~0.62), and Preop-IM plus Postop-IM (NMA: OR 0.09, 95% CI 0.02~0.36; cNMA: OR 0.11, 95% CI 0.03~0.37) demonstrated a clinical benefit by both NMA and cNMA when compared with Postop-SEN. The analysis of inconsistencies between direct and indirect evidence is demonstrated in Supplementary Figure S2(1–3), and no discrepancy was found between the direct and indirect evidence.

**Figure 3:**
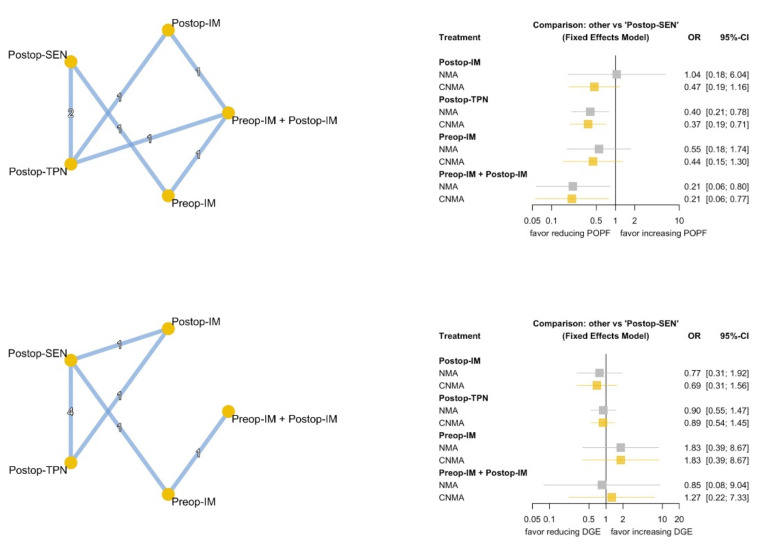
Network diagram and forest plot of NMA and cNMA for POPF and DGE. NMA: network meta-analysis; cNMA: component network meta-analysis; POPF: postoperative pancreatic fistula; DGE: delayed gastric emptying.

POPF and DGE are both important adverse outcomes of PD (Figure 3). Regarding POPF, Postop-TPN (NMA: OR 0.40, 95% CI 0.21~0.78; cNMA: OR 0.37, 95% CI 0.19~0.71) and Preop-IM plus Postop-IM (NMA: OR 0.21, 95% CI 0.06~0.80; cNMA: OR 0.21, 95% CI 0.06~0.77) had a clinical benefit when compared with Postop-SEN. In our analysis, none of the investigated nutrition therapies were superior to Postop-SEN in terms of DGE. The analysis of inconsistencies between direct and indirect evidence is demonstrated in Supplementary Figure S2(4,5), and no discrepancy was found between the direct and indirect evidence.

### 3.4. Secondary Outcomes

**Figure 4:**
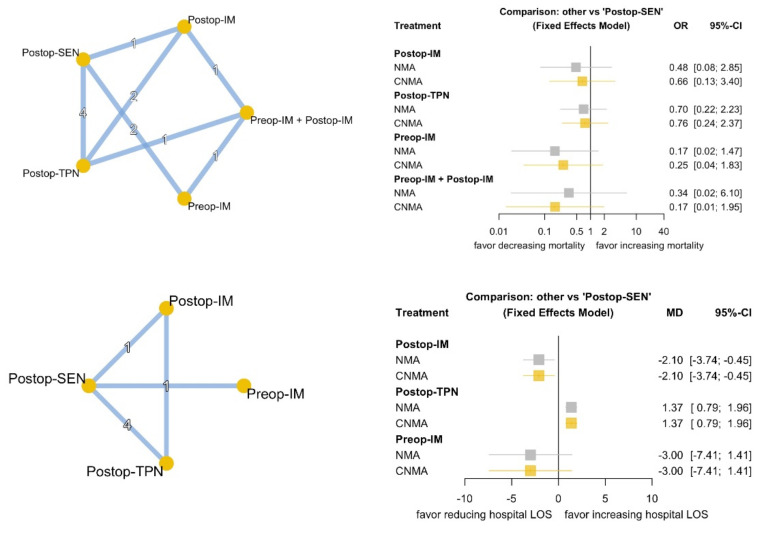
Network diagram and forest plot of NMA and cNMA for mortality and hospital LOS. NMA: network meta-analysis; cNMA: component network meta-analysis; LOS: length of stay.

None of the nutrition therapies significantly influenced mortality. Regarding the hospital LOS, Postop-TPN may prolong the LOS (MD 1.37, 95% CI 0.79~1.96), whereas Postop-IM can decrease the LOS (MD −2.10, 95% CI −3.74~−0.45). As only six studies had available LOS data, and no combinations of nutrition therapies were applied in these studies, the NMA and cNMA results were equivalent. The secondary outcome results are summarized in Figure 4. The analysis of inconsistencies between the direct and indirect evidence is demonstrated in Supplementary Figure S2(6,7), and no discrepancy was found between the direct and indirect evidence.

### 3.5. Relative Ranking of Nutrition Therapy

The SUCRA ranking of the different nutrition therapies is summarized in Supplementary Table S1(1,2). Of note, Preop-IM plus Postop-IM had a SUCRA value (0.9225) in relation to POPF incidence, indicating that this intervention provided the best clinical benefit for POPF prevention among the evaluated therapies.

### 3.6. Inconsistency and Publication Bias Assessment

The global inconsistency between the studies was well evaluated, and there was no significant heterogeneity among the outcomes with statistical significance (infectious complications, POPF, and LOS). The I^2^ was 0% for all aforementioned analyses with both NMA and cNMA (Supplementary Table S2). The results of the publication bias assessment revealed no significant bias, and the results regarding individual outcomes are summarized in Supplementary Figure S3(1–7).

### 3.7. Certainty of Evidence

The summary of GRADE recommendations for each outcome is provided in the Supplementary Materials (Supplementary Table S3(1–7)). Since not all of the enrolled studies were blinded, the level of certainty was downgraded at the beginning of the analysis. While some of the direct or indirect comparisons were at the “moderate” level, the certainty of NMA was downgraded further due to imprecision.

## 4. Discussion

PD has been recognized as one of the most complicated procedures in the field of gastrointestinal surgery. PD was first proposed in the early 20th century. Due to improved surgical techniques and postoperative care strategies, the mortality rate of patients treated with PD has decreased from 30% to 1% in high-volume institutions [24]. However, the morbidity rate remains as high as 30% [25]. In addition to improving delicate surgical techniques, the application of supportive perioperative management strategies is also necessary for improved patient outcomes. While early enteral nutrition has been suggested to improve immune function, reduce postoperative infection, and maintain intestinal barrier integrity in patients undergoing major abdominal surgery, adverse gastrointestinal effects (such as delayed gastric emptying, diarrhea, and abdominal discomfort) may prevent patients from receiving adequate enteral nutrition [26]. These adverse effects may be more obvious in patients undergoing PD than in those undergoing other gastrointestinal procedures. Therefore, hybrid nutritional interventions involving both enteral and parenteral routes with or without specialized formulas play a role in postoperative recovery, and several strategies for such treatment have been proposed and tested. In our report, relevant studies (RCTs only) were collected, and the optimal strategy for perioperative nutrition therapy for patients undergoing PD was determined. According to our results, the clinical outcomes affected by nutritional interventions were infectious complications and the occurrence of POPF. Three different nutritional interventions, Preop-IM, Preop-IM plus Postop-IM, and Preop-EN plus Postop-SEN, provided significant benefit in terms of reducing infectious complications. Preop-IM plus Postop-IM and Preop-EN plus Postop-SEN both had high SUCRA values (0.888 and 0.850, respectively), indicating that both of these interventions are superior to other management approaches. In fact, the IM formulas applied in our analyzed studies were all enteral. Therefore, the decrease in infectious complications observed may be related to the provision of enteral nutrition. Our analysis also revealed that Preop-IM plus Postop-IM and Postop-TPN provide clinical benefit in terms of reducing POPF. The SUCRA value of Preop-IM plus Postop-IM was high (0.923). Therefore, Preop-IM plus Postop-IM may be the optimal treatment for addressing infection complications and POPF occurrence.

NMA has the advantage of comparing multiple treatment options for a specific clinical circumstance. NMA is a special form of meta-regression that enables the simultaneous comparison of multiple treatments or interventions in a single study [27] and has been applied in medical research in recent decades. cNMA is an advanced application developed to explore the effects of different components of a complex treatment or intervention [13]. Examples of complex treatment evaluated in our enrolled trials were Preop-IM plus Postop-IM and Preop-EN plus Postop-SEN. Each treatment can be further separated into two components, and this methodology (cNMA) can improve the sensitivity and precision of the analysis. In recent decades, many physicians have focused on evaluating accumulated evidence in the field of post-PD nutrition therapy [28,29,30]. However, nutrition therapies are diverse and complicated in terms of route, regimen, and timing of intervention. Some evaluations have only demonstrated the result of systematic searching and did not include a synthesis of the evidence, and other studies have omitted evidence due to inconsistent design among studies. In our study, we accumulated evidence using a novel approach, namely, NMA, which is appropriate due to the complexity of post-PD nutrition therapy. In addition, the cNMA model was applied, which can analyze the precise effect of each component used in combined nutrition therapy approaches. Additionally, we observed that the effects of Preop-IM and Postop-IM were additive.

This study had several limitations. First, we did not include unpublished studies in the analysis. While we performed a thorough survey of published and relevant RCTs, we did not have access to unpublished data, and thus, we could not include information from these studies. Second, the most concerning issue in our eligible studies was bias from blindness inadequacies. Blindness-related bias can be divided into performance bias (patients and study personnel) and bias from the perspective of outcome measurement. Only one study fulfilled the blindness of outcome measurement criteria [20]. This issue may be inevitable for RCTs evaluating nutritional therapy since the formulas themselves have specific characteristics, and both patients and clinicians can distinguish different formulas even without additional information. This bias undoubtedly downgraded the strength of our results. Third, the number of individual studies enrolled in our analysis was relatively small (Table 1). While two studies enrolled over 200 subjects [6,16], most of the studies enrolled fewer than 50 subjects overall. The small number of subjects and events may have decreased the strength of the summarized evidence. Finally, the only IM used in the analyzed enrolled studies was IMPACT^®^. Therefore, the clinical benefit of other IMs, such as glutamine, fish oil, and nucleotides, should be further studied.

## 5. Conclusions

Our NMA and cNMA summarized the most relevant RCTs evaluating perioperative nutritional intervention for PD to date. Preop-IM plus Postop-IM may provide the best clinical benefit for patients undergoing PD to decrease infectious complications and POPF.

## Figures and Tables

**Table 1:** Summary of enrolled studies.

Author, Year	Country	Study Type	Patient	Treatment vs. Control	*n*	Age, Mean ± SD	Male, *n* (%)	Outcome	Brand of IM
	Immunonutrition vs. other								
Gianotti, 2000 [16]	Italy	RCT	PD or PPPD for pancreatic head or periampullary lesion	postop IM vs. SEN vs. postop TPN	71/73/68	61.1 ± 11.9/ 59.8 ± 12.2/ 60.2 ± 10.4	44 (62.0%)/ 47 (64.4%)/ 43 (63.2%)	mortality, complications, infectious complications, noninfectious complications, POPF, DGE, hospital LOS	IMPACT
Suzuki, 2010 [17]	Japan	RCT	PD or PPPD	preop IM + postop IM vs. postop IM vs. postop TPN	10/10/10	62.0 ± 4.0/ 61.0 ± 3.0/ 66.0 ± 3.0	7 (70%)/ 7 (70%)/ 4 (40%)	mortality, infectious complications, noninfectious complications, POPF	IMPACT
Aida, 2014 [18]	Japan	RCT	PD or PPPD	preop IM vs. SEN	25/25	66.4 ± 1.5/ 65.1 ± 1.9	20 (80%)/ 16 (64%)	mortality, complications, infectious complications, noninfectious complications, POPF, DGE	IMPACT
Hamza, 2015 [19]	United Kingdom	RCT	PD for periampullary cancer	preop IM + postop IM vs. preop EN + SEN	17/20	63.3 ± 3.2/ 66.8 ± 2.0	9 (52.9%)/ 11 (55%)	infectious complications, noninfectious complications	IMPACT
Gade, 2016 [20]	Denmark	RCT	PD and other surgery for pancreatic cancer	preop IM vs. SEN	19/16	66.8 ± 8.9/ 67.5 ± 7.5	12 (63.2%)/ 6 (37.5%)	mortality, hospital LOS	IMPACT
Miyauchi, 2019 [21]	Japan	RCT	PD or PPPD	preop IM + postop IM vs. preop IM	30/30	67.8 ± 9.3/ 67.6 ± 7.5	16 (53.3%)/ 18 (60%)	mortality, complications, infectious complications, noninfectious complications, POPF, DGE	IMPACT
	EN vs. TPN								
Liu, 2011 [22]	China	RCT	PD for pancreatic cancer	SEN vs. postop TPN	28/30	59.7 ± 11.2/ 60.5 ± 11.9	16 (57.1%)/ 17 (56.7%)	mortality, POPF, DGE, hospital LOS	-
Park, 2012 [23]	Korea	RCT	PD or PPPD	SEN vs. postop TPN	18/20	62.7 ± 10.3/ 61.3 ± 13.2	7 (38.9%)/ 12 (60%)	mortality, complications, infectious complications, noninfectious complications, POPF, DGE, hospital LOS	-
Perinel, 2016 [6]	France	RCT	PD or PPPD	SEN vs. postop TPN	103/101	65.46 ± 11.25/ 64.02 ± 9.9	39 (37.9%)/ 40 (39.6%)	mortality, complications, infectious complications, noninfectious complications, POPF, DGE, hospital LOS	-

## Data Availability

Not applicable.

## Supplementary Materials

The following are available online at https://www.mdpi.com/article/10.3390/nu13114049/s1, File S1: The detailed search strategy used for SR-NMA; Figure S1: Table summarizing the risk of bias; Figure S2 (1): Stepwise comparisons of different interventions and results of the SIDE approach for inconsistency assessment of direct and indirect evidence for complications. SIDE: Separating Indirect from Direct Evidence. (2): Stepwise comparisons of different interventions and results of the SIDE approach for inconsistency assessment of direct and indirect evidence for infectious complications. SIDE: Separating Indirect from Direct Evidence. (3): Stepwise comparisons of different interventions and results of the SIDE approach for inconsistency assessment of direct and indirect evidence for noninfectious complications. SIDE: Separating Indirect from Direct Evidence. (4): Stepwise comparisons of different interventions and results of the SIDE approach for inconsistency assessment of direct and indirect evidence for POPF. SIDE: Separating Indirect from Direct Evidence. (5): Stepwise comparisons of different interventions and results of the SIDE approach for inconsistency assessment of direct and indirect evidence for DGE. SIDE: Separating Indirect from Direct Evidence. (6): Stepwise comparisons of different interventions and results of the SIDE approach for inconsistency assessment of direct and indirect evidence for mortality. SIDE: Separating Indirect from Direct Evidence. (7): Stepwise comparisons of different interventions and results of the SIDE approach for inconsistency assessment of direct and indirect evidence for hospital LOS. SIDE: Separating Indirect from Direct Evidence; Figure S3 (1): Funnel plot showing general complications. (2): Funnel plot showing infectious complications. (3): Funnel plot showing noninfectious complications. (4): Funnel plot showing POPF outcomes. (5): Funnel plot showing DGE outcomes. (6): Funnel plot showing mortality outcomes. (7): Funnel plot showing hospital LOS; Table S1 (1): SUCRA ranking of complications. (2): SUCRA ranking of POPF, DGE, hospital LOS, and mortality. Table S2: Summary of inconsistency. Table S3 (1): GRADE recommendation for all complication. (2): GRADE recommendation for infectious complication. (3): GRADE recommendation for noninfectious complication. (4): GRADE recommendation for POPF. (5): GRADE recommendation for DGE. (6): GRADE recommendation for mortality. (7): GRADE recommendation for hospital LOS.
