# Supplementary material for: Optimal Perioperative Nutrition Therapy for Patients Undergoing Pancreaticoduodenectomy: A Systematic Review with a Component Network Meta-Analysis
Source: Nutrients. 2021 Nov 12;13(11):4049. doi: 10.3390/nu13114049 (PMC8620471; doi:10.3390/nu13114049)
Supplement: Supplementary file 1 [file nutrients-13-04049-s001.zip › nutrients-1448740-supplementary.pdf]

Supplementary Document

1.Cochrane library

Date Run: 20/07/2021 15:14:03

Comment:

ID	Search	Hits
#1	MeSH descriptor: [Pancreatic Neoplasms] explode all trees	1835
#2	MeSH descriptor: [Nutrition Therapy] explode all trees	9688
#3	immunonutrition	341
#4	MeSH descriptor: [Pancreaticoduodenectomy] explode all trees	277
#5	MeSH descriptor: [Glutamine] explode all trees	665
#6	MeSH descriptor: [Arginine] explode all trees	1494
#7	MeSH descriptor: [Fatty Acids, Omega-3] explode all trees	3197
#8	MeSH descriptor: [Nucleotides] explode all trees	2675
#9	MeSH descriptor: [Enteral Nutrition] explode all trees	1896
#10	MeSH descriptor: [Parenteral Nutrition, Total] explode all trees	766
#11	(#2 or #3 or #5 or #6 or #7 or #8 or #9 or #10) and (#1 or #4)	64

2.Embase

No.	Query Results	Results	Date
#2.	(('pancreas cancer'/exp OR 'pancreaticoduodenectomy'/exp OR 'pylorus preserving pancreaticoduodenectomy'/exp) AND ('diet therapy'/exp OR 'glutamine'/exp OR 'arginine'/exp OR 'omega 3 fatty acid'/exp OR 'nucleotide'/exp OR 'enteric feeding'/exp OR 'total parenteral nutrition'/exp)) AND ('clinical trial'/de OR 'randomized controlled trial'/de OR 'randomized controlled trial topic'/de) AND ('article'/it OR 'article in press'/it)	245	20 Jul 2021
#1.	('pancreas cancer'/exp OR 'pancreaticoduodenectomy'/exp OR 'pylorus preserving pancreaticoduodenectomy'/exp) AND ('diet therapy'/exp OR 'glutamine'/exp OR 'arginine'/exp OR 'omega 3 fatty acid'/exp OR 'nucleotide'/exp OR 'enteric feeding'/exp OR 'total parenteral nutrition'/exp)	5,064	20 Jul 2021

3.Pubmed

Query	((pancreas neoplasm[MeSH Terms]) OR (pancreaticoduodenectomy[MeSH Terms]) OR (Whipple) OR (Pylorus preserving pancreaticoduodenectomy)) AND ((enteral feeding[MeSH Terms]) OR (total parenteral nutrition[MeSH Terms]) OR (nutrition therapy[MeSH Terms]) OR (immunonutrition) OR (immune modulating) OR (glutamine) OR (arginine) OR (nucleotide) OR (omega 3 fatty acids[MeSH Terms]) OR (fish oils[MeSH Terms]))
Filters	Clinical Study, Clinical Trial, Randomized Controlled Trial
Search Details	(("pancreatic neoplasms"[MeSH Terms] OR "pancreaticoduodenectomy"[MeSH Terms] OR ("whipple"[All Fields] OR "whipple s"[All Fields] OR "whipples"[All Fields]) OR (("pylorus"[MeSH Terms] OR "pylorus"[All Fields]) AND ("preservation, biological"[MeSH Terms] OR ("preservation"[All Fields] AND "biological"[All Fields]) OR "biological preservation"[All Fields] OR "preservation"[All Fields] OR "preserved"[All Fields] OR "preservations"[All Fields] OR "preserve"[All Fields] OR "preserves"[All Fields] OR "preserving"[All Fields]) AND ("pancreaticoduodenectomy"[MeSH Terms] OR "pancreaticoduodenectomy"[All Fields] OR "pancreaticoduodenectomies"[All Fields]))) AND ("enteral nutrition"[MeSH Terms] OR "parenteral nutrition, total"[MeSH Terms] OR ("nutritional support"[MeSH Terms] OR "nutrition therapy"[MeSH Terms]) OR ("immunonutrition"[All Fields] OR "immunonutritional"[All Fields]) OR (("immune"[All Fields] OR "immuned"[All Fields] OR "immunes"[All Fields] OR "immunisation"[All Fields] OR "vaccination"[MeSH Terms] OR "vaccination"[All Fields] OR "immunization"[All Fields] OR "immunization"[MeSH Terms] OR "immunisations"[All Fields] OR "immunizations"[All Fields] OR "immunise"[All Fields] OR "immunised"[All Fields] OR "immuniser"[All Fields] OR "immunisers"[All Fields] OR "immunising"[All Fields] OR "immunities"[All Fields] OR "immunity"[MeSH Terms] OR "immunity"[All Fields] OR "immunization s"[All Fields] OR "immunize"[All Fields] OR "immunized"[All Fields] OR "immunizer"[All Fields] OR "immunizers"[All Fields] OR "immunizes"[All Fields] OR "immunizing"[All Fields]) AND ("modulate"[All Fields] OR "modulated"[All Fields] OR "modulates"[All Fields] OR "modulating"[All Fields] OR "modulation"[All Fields] OR "modulations"[All Fields] OR "modulator"[All Fields] OR "modulators"[All Fields])) OR ("glutamin"[All Fields] OR "glutamine"[MeSH Terms] OR "glutamine"[All Fields] OR "glutamine s"[All Fields] OR "glutamines"[All Fields]) OR ("arginin"[All Fields] OR "arginine"[MeSH Terms] OR "arginine"[All Fields] OR "arginine s"[All Fields] OR "arginines"[All Fields] OR "argininic"[All Fields]) OR ("nucleotid"[All Fields] OR "nucleotides"[MeSH Terms] OR "nucleotides"[All Fields] OR "nucleotide"[All Fields] OR "nucleotidic"[All Fields]) OR "fatty acids, omega 3"[MeSH Terms] OR "fish oils"[MeSH Terms])) AND (clinicalstudy[Filter] OR clinicaltrial[Filter] OR randomizedcontrolledtrial[Filter])
Results	141

4.ClinicalTrial.gov

nutrition therapy OR immunonutrition | Pancreatic Neoplasms OR pancreatic cancers OR pancreaticoduodenectomy

Figure S1: Table summarizing the risk of bias.

	Random sequence generation (selection bias)	Allocation concealment (selection bias)	Blinding of participants and personnel (performance bias)	Blinding of outcome assessment (detection bias)	Incomplete outcome data (attrition bias)	Selective reporting (reporting bias)	Other bias
Aida 2014	−	+	?	?	+	−	+
Gade 2016	?	+	?	+	?	+	+
Gianotti 2000	?	+	?	?	+	+	+
Hamza 2015	+	+	?	?	?	+	+
Liu 2011	−	−	?	?	−	+	+
Miyauchi 2019	+	+	?	?	−	+	+
Park 2012	?	+	?	?	?	+	+
Perinel 2016	+	+	?	?	?	+	+
Suzuki 2010	?	+	?	?	+	+	+

Figure S2–1: Stepwise comparisons of different interventions and results of the SIDE approach for inconsistency assessment of direct and indirect evidence for complications. SIDE: Separating Indirect from Direct Evidence.

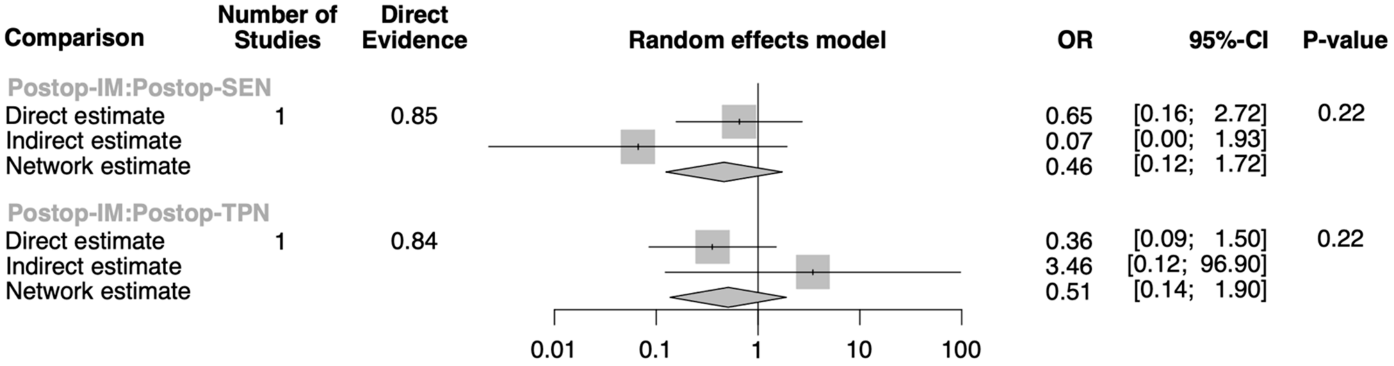

Figure S2–2: Stepwise comparisons of different interventions and results of the SIDE approach for inconsistency assessment of direct and indirect evidence for infectious complications. SIDE: Separating Indirect from Direct Evidence.

Comparison	Number of Studies	Direct Evidence	OR	95%-CI	P-value
**Postop-IM:Postop-SEN**					
Direct estimate	1	0.68	0.52	[0.18; 1.49]	0.81
Indirect estimate			0.66	[0.14; 3.07]	
Network estimate			0.56	[0.23; 1.34]	
**Postop-IM:Postop-TPN**					
Direct estimate	2	0.91	0.43	[0.18; 1.03]	0.47
Indirect estimate			1.25	[0.08; 20.46]	
Network estimate			0.47	[0.20; 1.09]	
**Postop-IM:Preop-IM + Postop-IM**					
Direct estimate	1	0.41	13.50	[1.20; 152.21]	0.43
Indirect estimate			3.83	[0.51; 28.95]	
Network estimate			6.43	[1.36; 30.35]	
**Postop-TPN:Postop-SEN**					
Direct estimate	3	0.97	1.20	[0.76; 1.90]	0.79
Indirect estimate			0.82	[0.05; 13.22]	
Network estimate			1.19	[0.75; 1.87]	
**Preop-IM:Postop-SEN**					
Direct estimate	1	0.84	0.26	[0.08; 0.85]	0.68
Indirect estimate			0.14	[0.01; 2.02]	
Network estimate			0.23	[0.08; 0.69]	
**Postop-TPN:Preop-IM + Postop-IM**					
Direct estimate	1	0.35	13.50	[1.20; 152.21]	0.99
Indirect estimate			13.68	[2.29; 81.89]	
Network estimate			13.62	[3.23; 57.43]	
**Preop-IM:Preop-IM + Postop-IM**					
Direct estimate	1	0.79	2.36	[0.63; 8.92]	0.68
Indirect estimate			4.39	[0.32; 59.40]	
Network estimate			2.69	[0.82; 8.76]	

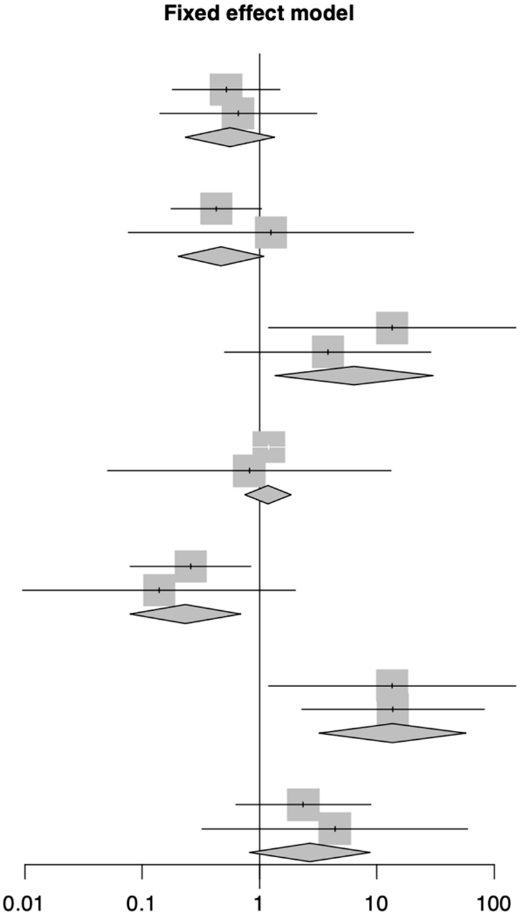

Figure S2–3: Stepwise comparisons of different interventions and results of the SIDE approach for inconsistency assessment of direct and indirect evidence for noninfectious complications. SIDE: Separating Indirect from Direct Evidence.

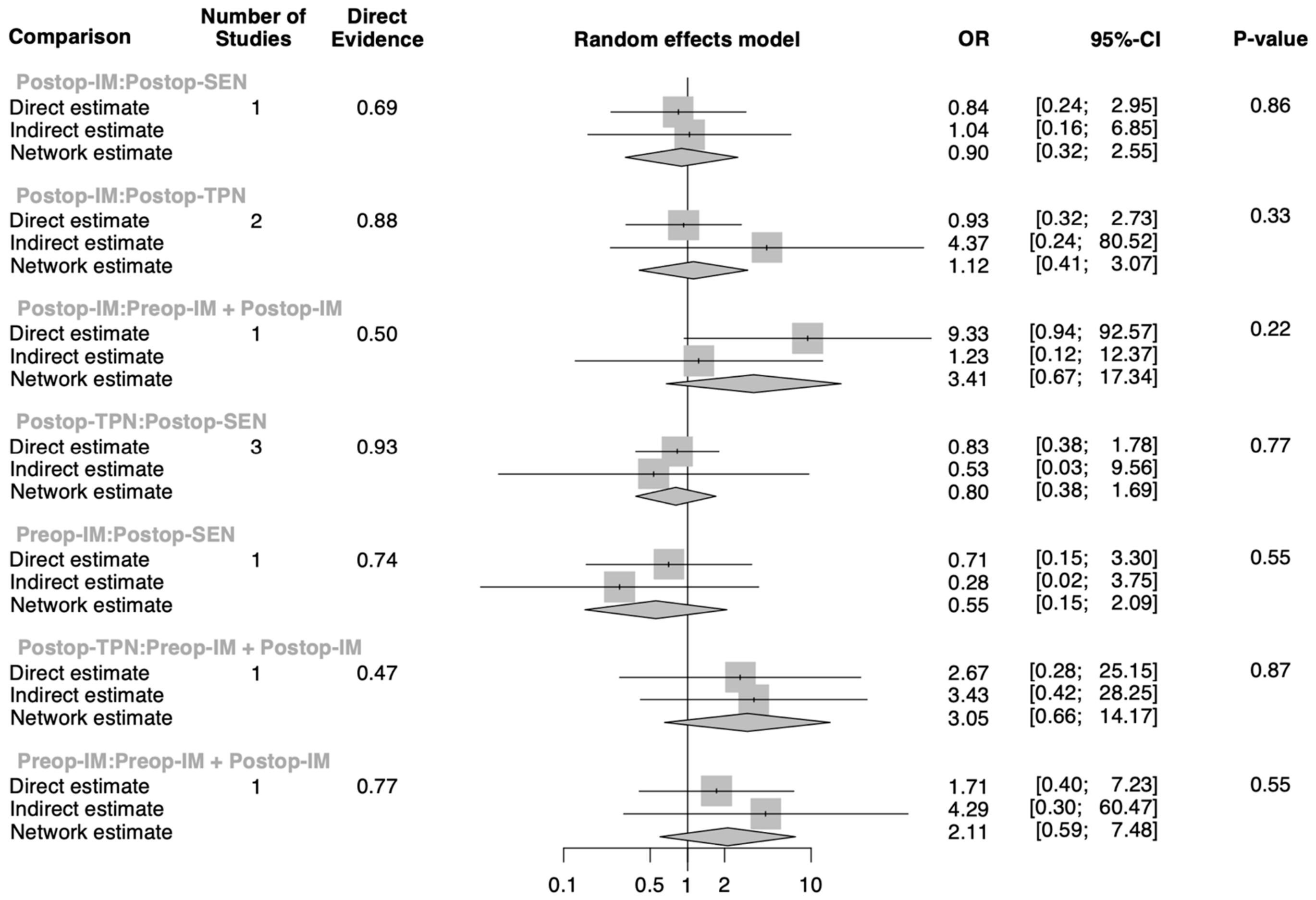

Figure S2–4: Stepwise comparisons of different interventions and results of the SIDE approach for inconsistency assessment of direct and indirect evidence for POPF. SIDE: Separating Indirect from Direct Evidence.

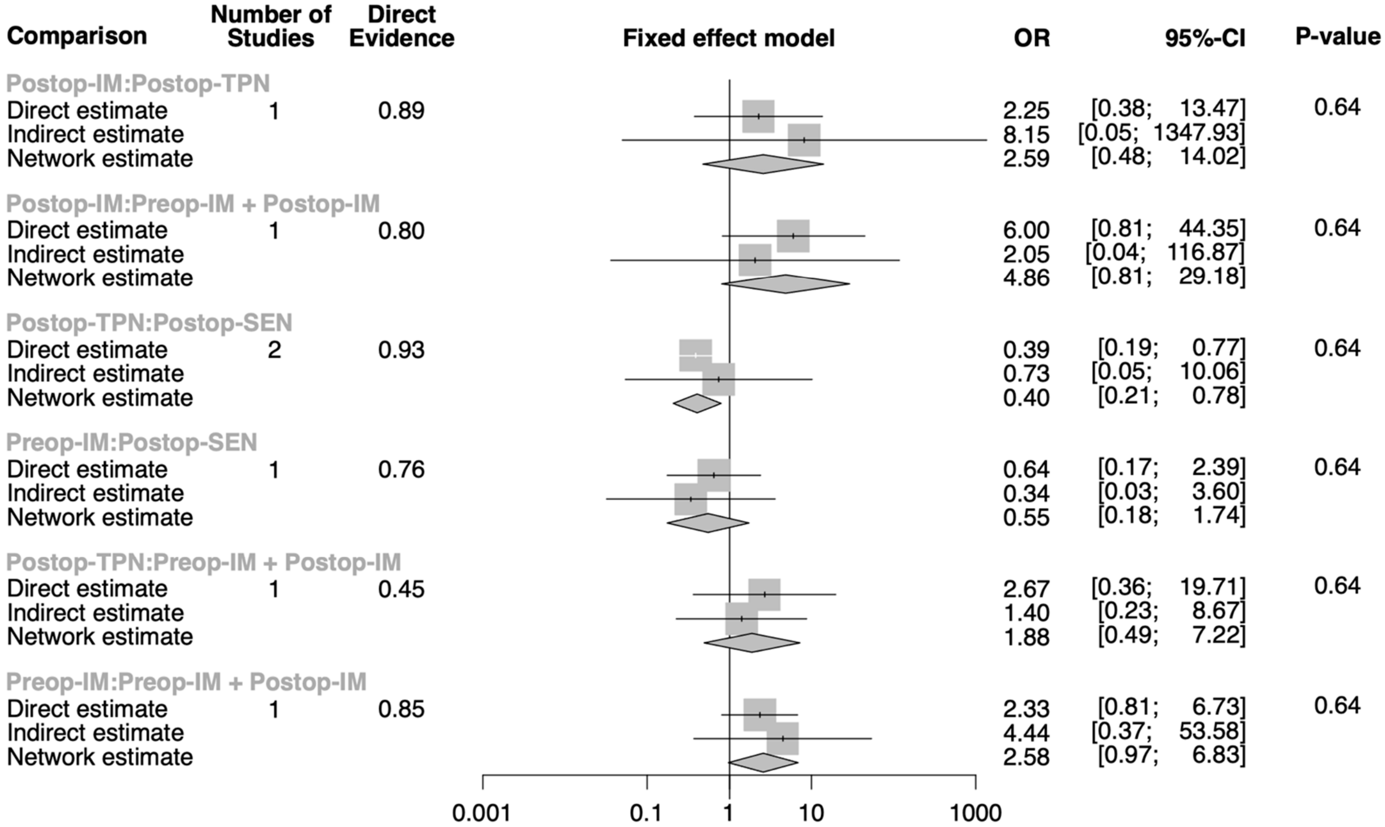

Figure S2–5: Stepwise comparisons of different interventions and results of the SIDE approach for inconsistency assessment of direct and indirect evidence for DGE. SIDE: Separating Indirect from Direct Evidence.

Comparison	Number of Studies	Direct Evidence	OR	95%-CI	P-value
**Postop-IM:Postop-SEN**					
Direct estimate	1	0.82	0.90	[0.33; 2.49]	0.47
Indirect estimate			0.37	[0.04; 3.20]	
Network estimate			0.77	[0.31; 1.92]	
**Postop-IM:Postop-TPN**					
Direct estimate	1	0.84	0.74	[0.27; 1.99]	0.47
Indirect estimate			1.85	[0.19; 17.88]	
Network estimate			0.85	[0.34; 2.13]	

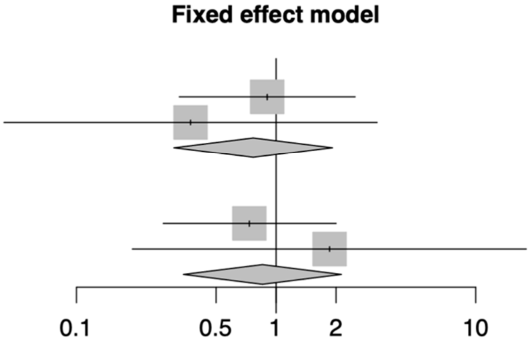

Figure S2–6: Stepwise comparisons of different interventions and results of the SIDE approach for inconsistency assessment of direct and indirect evidence for mortality. SIDE: Separating Indirect from Direct Evidence.

Comparison	Number of Studies	Direct Evidence	OR	95%-CI	P-value
**Postop-IM:Postop-SEN**					
Direct estimate	1	0.54	2.09	[0.19; 23.54]	0.08
Indirect estimate			0.09	[0.01; 1.19]	
Network estimate			0.48	[0.08; 2.85]	
**Postop-IM:Postop-TPN**					
Direct estimate	2	0.95	0.52	[0.11; 2.56]	0.15
Indirect estimate			76.51	[0.10; 60614.91]	
Network estimate			0.68	[0.15; 3.21]	
**Postop-IM:Preop-IM + Postop-IM**					
Direct estimate	1	0.55	1.00	[0.02; 55.52]	0.80
Indirect estimate			2.20	[0.03; 185.70]	
Network estimate			1.43	[0.07; 28.02]	
**Postop-TPN:Postop-SEN**					
Direct estimate	4	0.96	0.75	[0.23; 2.43]	0.58
Indirect estimate			0.13	[0.00; 58.46]	
Network estimate			0.70	[0.22; 2.23]	
**Preop-IM:Postop-SEN**					
Direct estimate	2	0.84	0.13	[0.01; 1.39]	0.60
Indirect estimate			0.63	[0.00; 143.28]	
Network estimate			0.17	[0.02; 1.47]	
**Postop-TPN:Preop-IM + Postop-IM**					
Direct estimate	1	0.51	1.00	[0.02; 55.52]	0.61
Indirect estimate			4.47	[0.08; 264.81]	
Network estimate			2.09	[0.12; 36.58]	
**Preop-IM:Preop-IM + Postop-IM**					
Direct estimate	1	0.56	1.00	[0.02; 52.06]	0.60
Indirect estimate			0.20	[0.00; 16.95]	
Network estimate			0.49	[0.03; 9.39]	

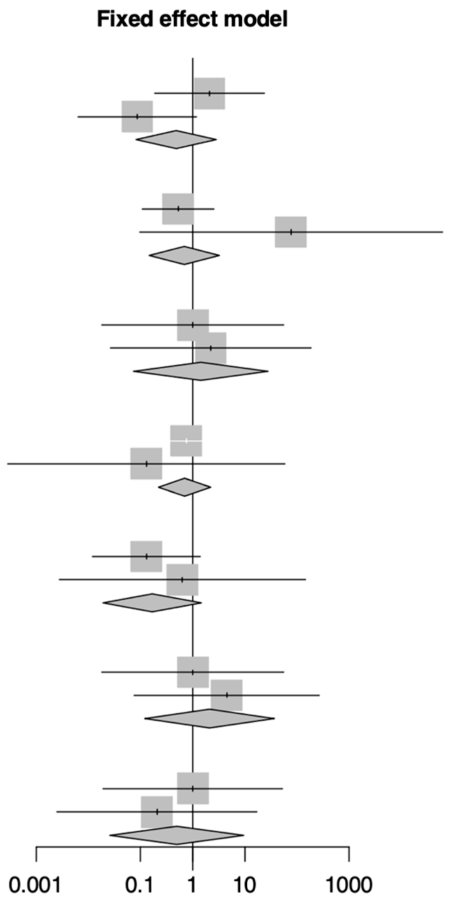

Figure S2–7: Stepwise comparisons of different interventions and results of the SIDE approach for inconsistency assessment of direct and indirect evidence for hospital LOS. SIDE: Separating Indirect from Direct Evidence.

Comparison	Number of Studies	Direct Evidence	Fixed effect model	MD	95%-CI	P-value
Postop-IM:Postop-SEN						
Direct estimate	1	0.77		-1.90	[-3.78; -0.02]	0.67
Indirect estimate				-2.74	[-6.15; 0.66]	
Network estimate				-2.10	[-3.74; -0.45]	
Postop-IM:Postop-TPN						
Direct estimate	1	0.70		-3.70	[-5.67; -1.73]	0.67
Indirect estimate				-2.92	[-5.96; 0.13]	
Network estimate				-3.47	[-5.12; -1.81]	

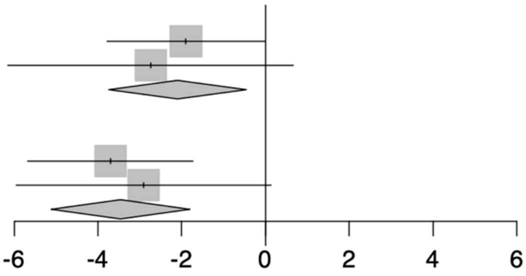

Figure S3–1: Funnel plot showing general complications.

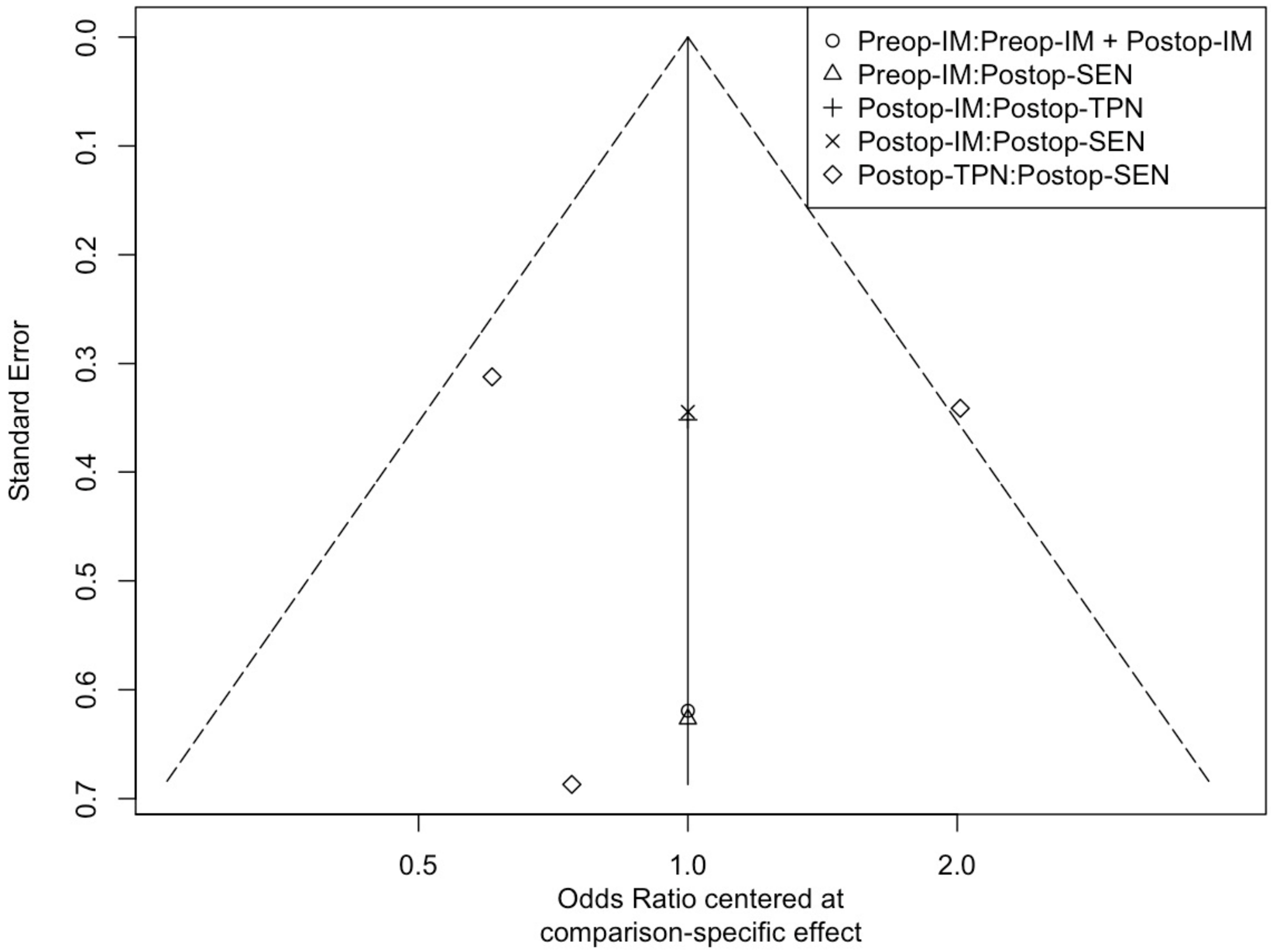

Figure S3–2: Funnel plot showing infectious complications.

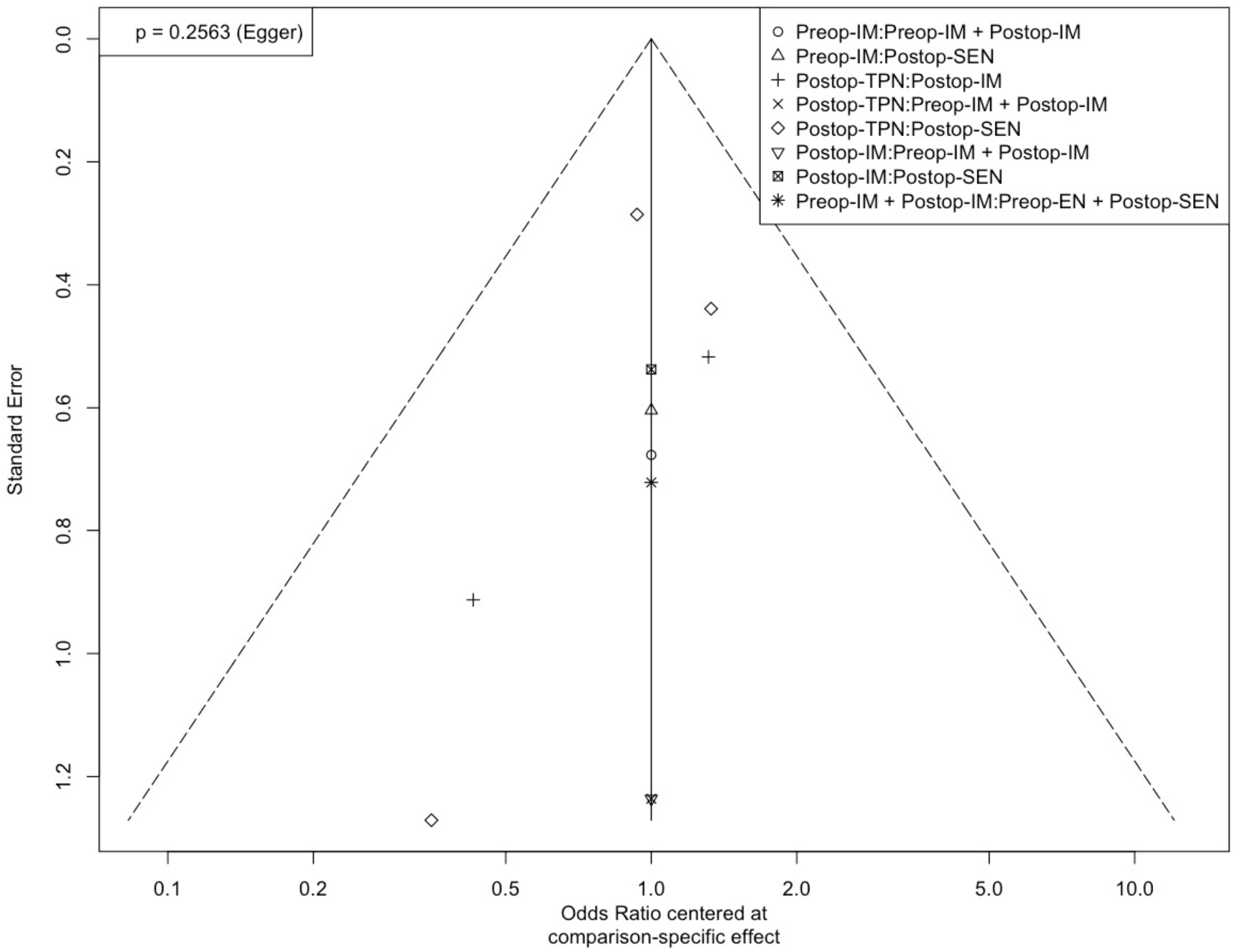

Figure S3–3: Funnel plot showing noninfectious complications.

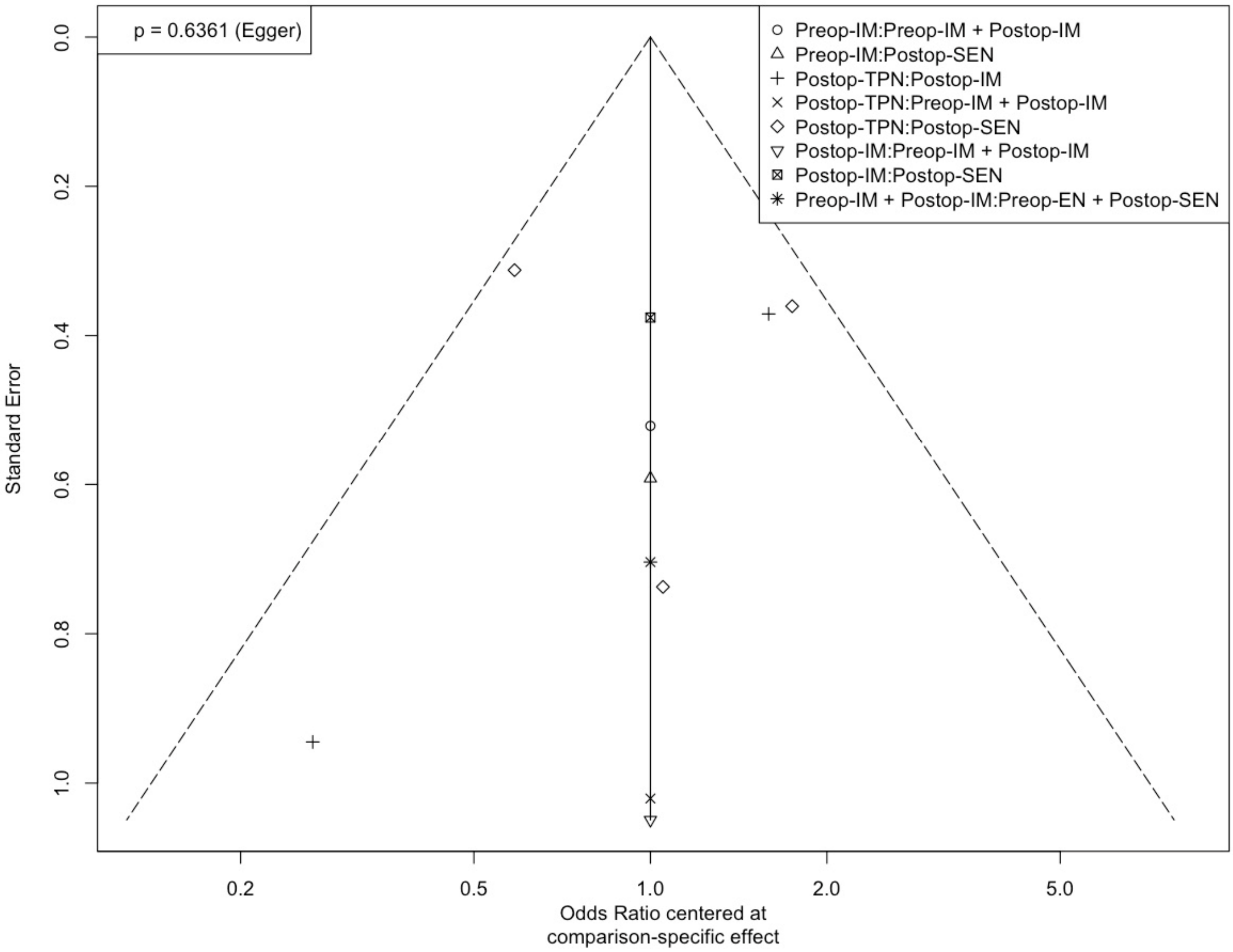

Figure S3–4: Funnel plot showing POPF outcomes.

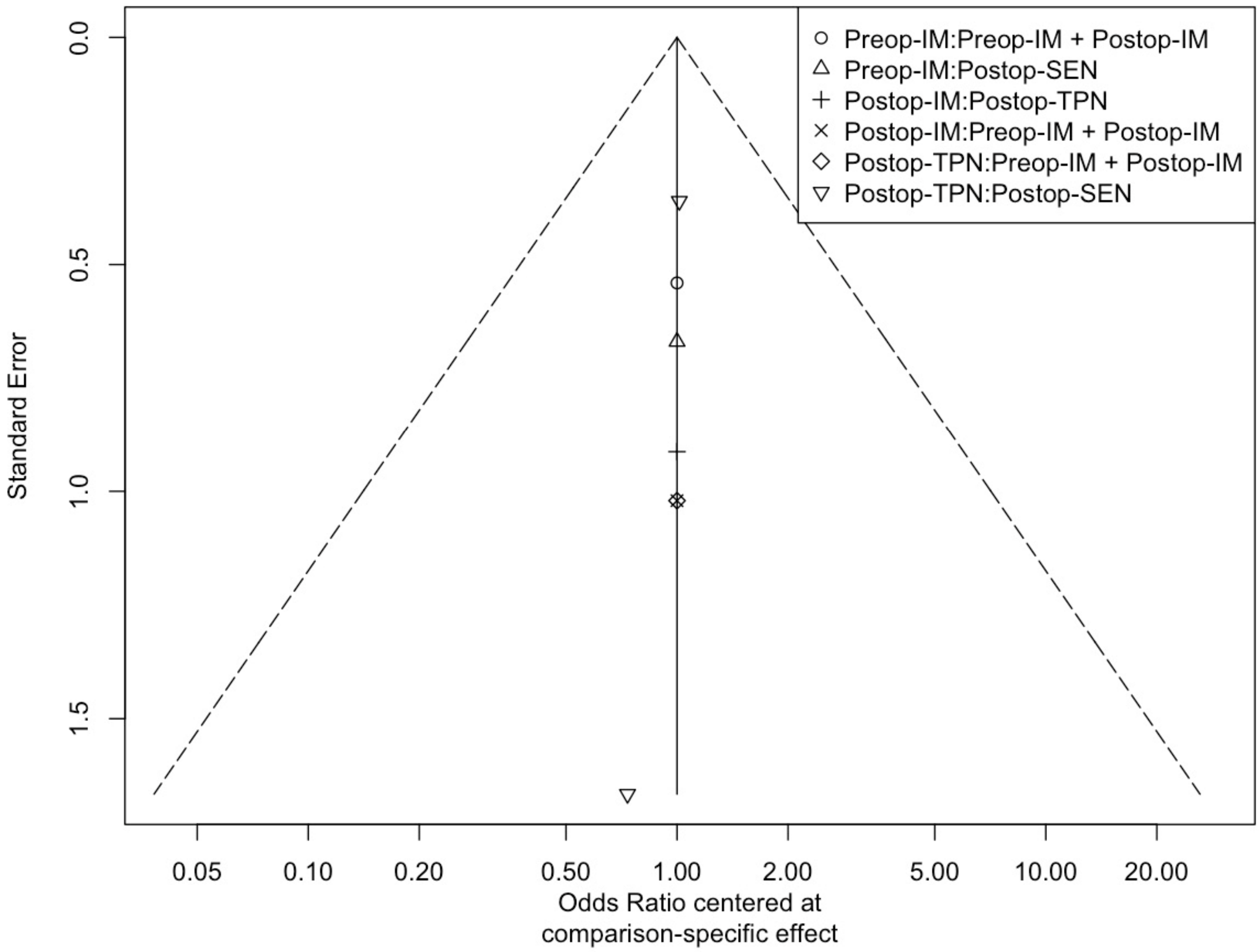

Figure S3–5: Funnel plot showing DGE outcomes.

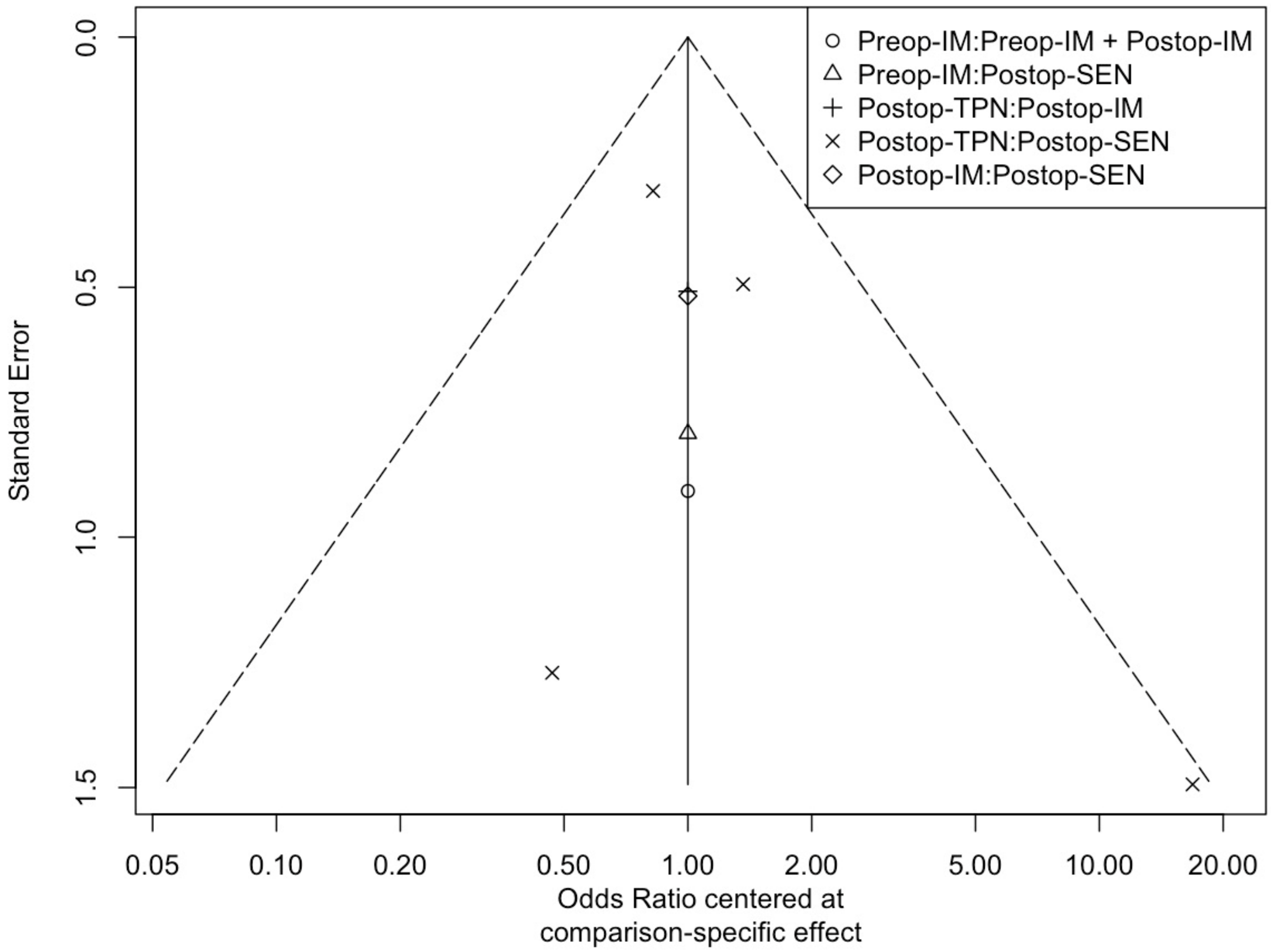

Figure S3–6: Funnel plot showing mortality outcomes.

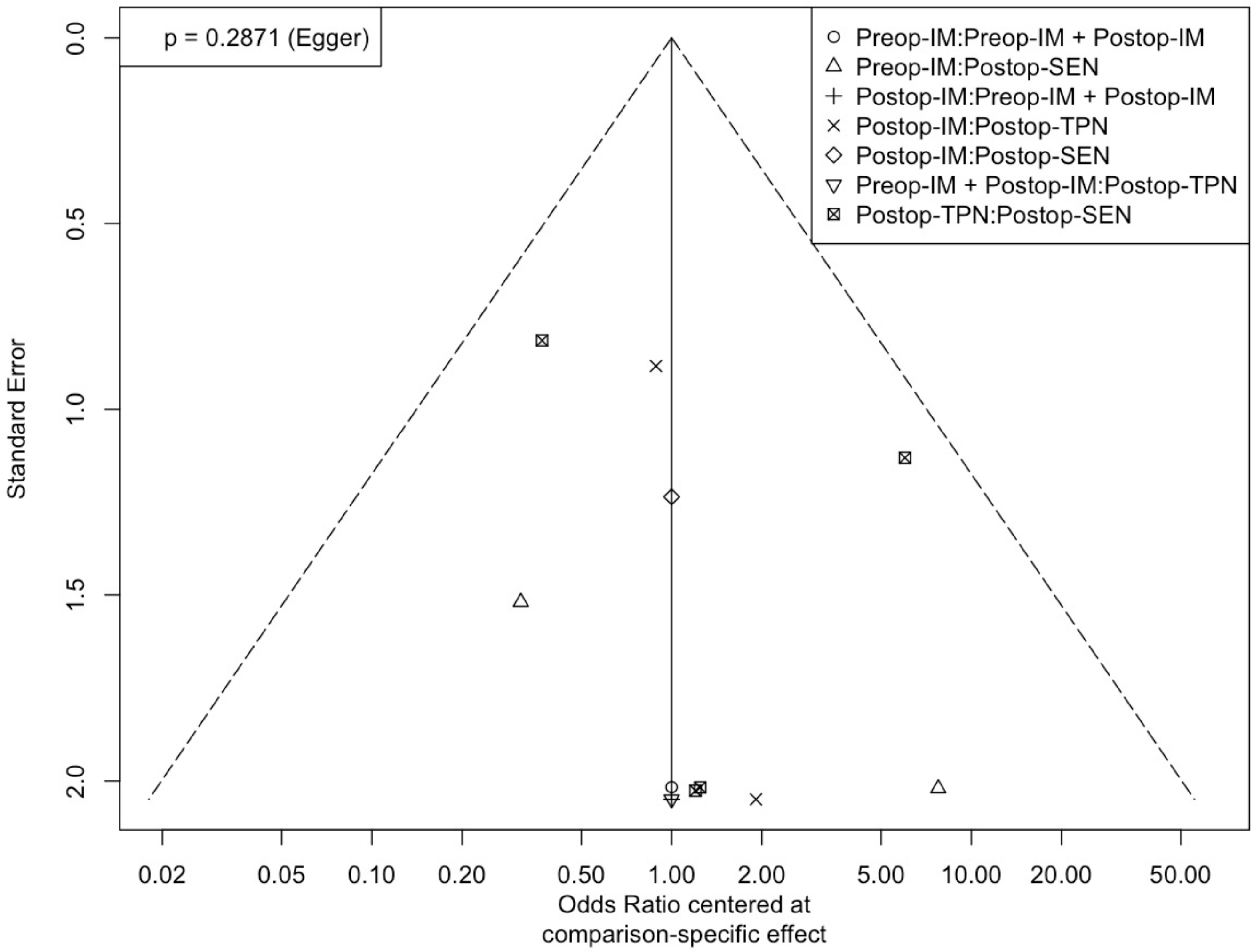

Figure S3–7: Funnel plot showing hospital LOS.

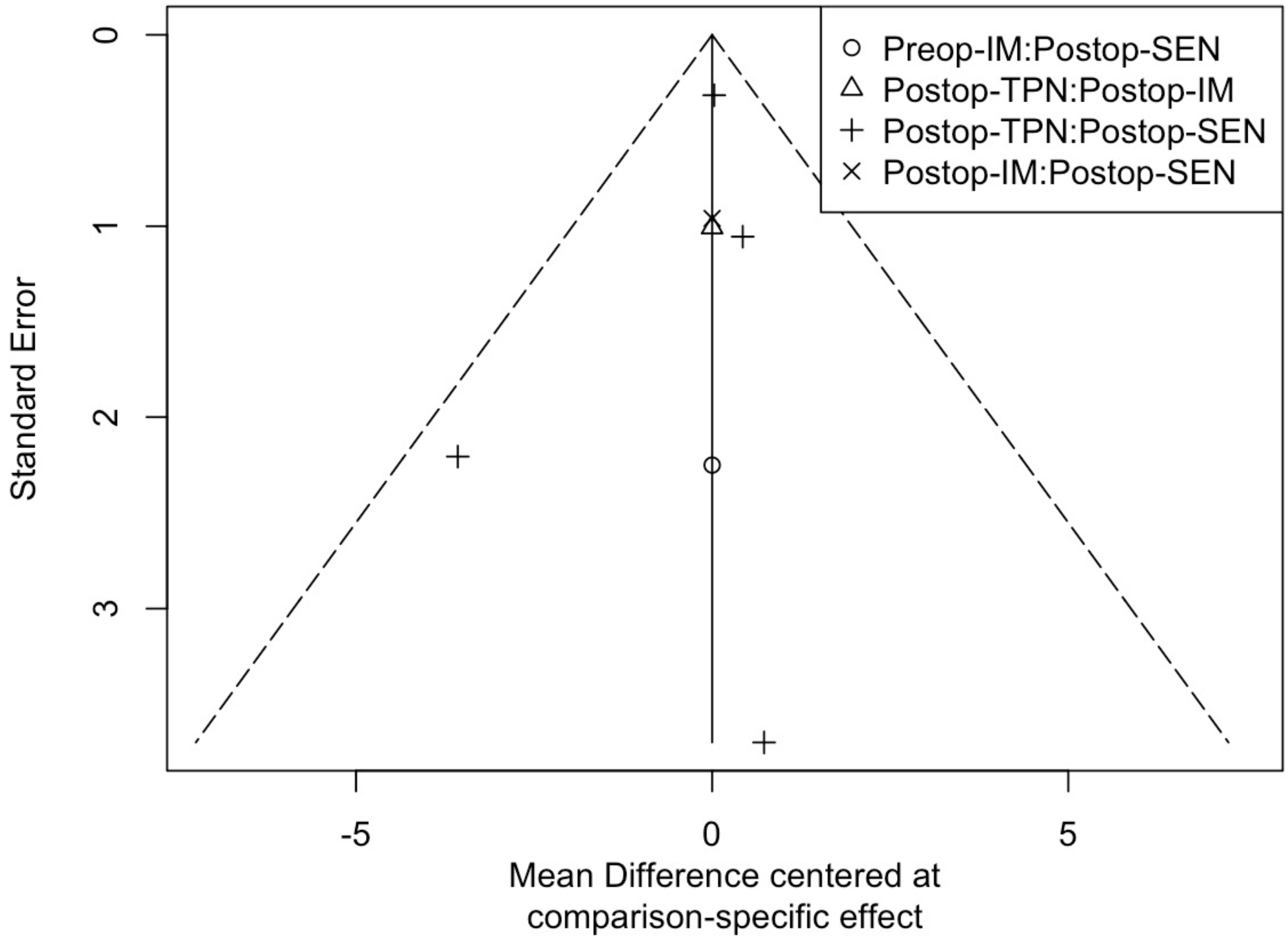

Supplementary Table S1–1

	SUCRA of complication		SUCRA of infectious complication		SUCRA of noninfectious complication
postop EIM	0.8325	preop EIM supplementation + postop EIM	0.888	preop EN supplementation + SEN	0.892
preop EIM supplementation + postop EIM	0.6025	preop EN supplementation + SEN	0.850	preop EIM supplementation + postop EIM	0.778
postop TPN	0.4250	preop EIM supplementation	0.614	preop EIM supplementation	0.528
SEN	0.3625	postop EIM	0.424	postop TPN	0.330
preop EIM supplementation	0.2775	SEN	0.158	postop EIM	0.268
		postop TPN	0.066	SEN	0.204

Supplementary Table S1–2

	SUCRA of POPF		SUCRA of DGE		SUCRA of hospital LOS		SUCRA of mortality
preop EIM supplementation + postop EIM	0.9225	postop EIM	0.7250	preop EIM supplementation	0.87	preop EIM supplementation	0.8175
postop TPN	0.6600	postop TPN	0.5225	postop EIM	0.75	preop EIM supplementation + postop EIM	0.6600
preop EIM supplementation	0.5000	preop EIM supplementation + postop EIM	0.5025	SEN	0.37	postop EIM	0.4075
SEN	0.2425	SEN	0.4200	postop TPN	0.01	postop TPN	0.3275
postop EIM	0.1750	preop EIM supplementation	0.3300			SEN	0.2875

Supplementary Table S2

	Enrolled study	Model	I^2	Tau
NMA				
Complication	5	REM	71.5%	0.64
Infectious complication	7	FEM	0%	0
Noninfectious complication	7	REM	50.6%	0.52
POPF	5	FEM	0%	0
DGE	6	FEM	36.5%	0.48
Mortality	8	FEM	0%	0
Hospital LOS	5	FEM	0%	0
cNMA				
Complication	5	REM	58.4%	0.51
Infectious complication	7	FEM	0%	0
Noninfectious complication	7	REM	42.1%	0.43
POPF	5	FEM	0%	0
DGE	6	FEM	19.5%	0.32
Mortality	8	FEM	0%	0
Hospital LOS	5	FEM	0%	0
REM, random effects model; FEM, fixed effects model				

Supplementary Table S3–1

Comparisons (study)	Direct		Indirect		Network meta-analysis	
	95% CI	Rating of direct evidence	95% CI	Rating of indirect evidence	95% CI	Certainty of evidence
Postop-IM : Postop-TPN	0.3574 (0.0853 ~ 1.4975)	Moderate ⊕⊕⊕◯	3.4584 (0.1234 ~ 96.9040)	Moderate ⊕⊕⊕◯	0.5092 (0.1366 ~ 1.8989)	Low* ⊕⊕◯◯
Postop-IM : Preop-IM			0.2596 (0.0289 ~ 2.3274)	Not estimate	0.2596 (0.0289 ~ 2.3274)	Not estimate
Postop-IM : Preop-IM + Postop-IM			0.7514 (0.0455 ~ 12.4045)	Not estimate	0.7514 (0.0455 ~ 12.4045)	Not estimate
Postop-IM : Postop-SEN	0.6543 (0.1572 ~ 2.7223)	Moderate ⊕⊕⊕◯	0.0661 (0.0023 ~ 1.9315)	Moderate ⊕⊕⊕◯	0.4624 (0.1243 ~ 1.7193)	Low* ⊕⊕◯◯
Postop-TPN : Preop-IM			0.5097 (0.0716 ~ 3.6310)	Low ⊕⊕◯◯	0.5097 (0.0716 ~ 3.6310)	Very low* ⊕◯◯◯
Postop-TPN : Preop-IM + Postop-IM			1.4755 (0.1066 ~ 20.4267)	Not estimate	1.4755 (0.1066 ~ 20.4267)	Not estimate
Postop-TPN : Postop-SEN	0.9080 (0.3779 ~ 2.1815)	Moderate ⊕⊕⊕◯			0.9080 (0.3779 ~ 2.1815)	Low* ⊕⊕◯◯
Preop-IM : Preop-IM + Postop-IM	2.8947 (0.5048 ~ 16.6012)	Low ⊕⊕◯◯			2.8947 (0.5048 ~ 16.6012)	Very low* ⊕◯◯◯
Preop-IM : Postop-SEN	1.7812 (0.3074 ~ 10.3209)	Low ⊕⊕◯◯			1.7813 (0.3074 ~ 10.3209)	Very low* ⊕◯◯◯
Preop-IM + Postop-IM : Postop-SEN			0.6153 (0.0517 ~ 7.3282)	Low ⊕⊕◯◯	0.6153 (0.0517 ~ 7.3282)	Very low* ⊕◯◯◯
*, down rating due to imprecision						

Supplementary Table S3–2

Comparisons (study)	Direct		Indirect		Network meta-analysis	
	95% CI	Rating of direct evidence	95% CI	Rating of indirect evidence	95% CI	Certainty of evidence
Postop-IM : Postop-TPN	0.4282 (0.1773 ~ 1.0346)	Moderate ⊕⊕⊕◯	1.2533 (0.0768 ~ 20.4554)	Moderate ⊕⊕⊕◯	0.4721 (0.2036 ~ 1.0947)	Low* ⊕⊕◯◯
Postop-IM : Preop-IM			2.3936 (0.6309 ~ 9.0815)	Low ⊕⊕◯◯	2.3936 (0.6309 ~ 9.0815)	Very low* ⊕◯◯◯
Postop-IM : Preop-IM + Postop-IM	13.5000 (1.1973 ~ 152.2113)	Moderate ⊕⊕⊕◯	3.8325 (0.5073 ~ 28.9518)	Moderate ⊕⊕⊕◯	6.4274 (1.3609 ~ 30.3549)	Low* ⊕⊕◯◯
Postop-IM : Preop EN + Postop-SEN			6.2489 (0.7647 ~ 51.0651)	Moderate ⊕⊕⊕◯	6.2489 (0.7647 ~ 51.0651)	Low* ⊕⊕◯◯
Postop-IM : Postop-SEN	0.5203 (0.1814 ~ 1.4925)	Moderate ⊕⊕⊕◯	0.6577 (0.1410 ~ 3.0668)	Moderate ⊕⊕⊕◯	0.5607 (0.2350 ~ 1.3378)	Low* ⊕⊕◯◯
Postop-TPN : Preop-IM			5.0705 (1.6056 ~ 16.0125)	Low ⊕⊕◯◯	5.0705 (1.6056 ~ 16.0125)	Very low* ⊕◯◯◯
Postop-TPN : Preop-IM + Postop-IM	13.5000 (1.1973 ~ 152.2113)	Moderate ⊕⊕⊕◯	13.6789 (2.2850 ~ 81.8861)	Moderate ⊕⊕⊕◯	13.6155 (3.2279 ~ 57.4308)	Low* ⊕⊕◯◯
Postop-TPN : Preop EN + Postop-SEN			13.2373 (1.7584 ~ 99.6496)	Moderate ⊕⊕⊕◯	13.2373 (1.7584 ~ 99.6496)	Low* ⊕⊕◯◯
Postop-TPN : Postop-SEN	1.1998 (0.7566 ~ 1.9029)	Moderate ⊕⊕⊕◯	0.8206 (0.0509 ~ 13.2197)	Moderate ⊕⊕⊕◯	1.1877 (0.7536 ~ 1.8719)	Low* ⊕⊕◯◯
Preop-IM : Preop-IM + Postop-IM	2.3636 (0.6265 ~ 8.9169)	Low ⊕⊕◯◯	4.3884 (0.3242 ~ 59.3975)	Not estimate	2.6853 (0.8227 ~ 8.7649)	Very low* ⊕◯◯◯
Preop-IM : Preop EN + Postop-SEN			2.6107 (0.4127 ~ 16.5137)	Low ⊕⊕◯◯	2.6107 (0.4127 ~ 16.5137)	Very low* ⊕◯◯◯
Preop-IM : Postop-SEN	0.2593 (0.0793 ~ 0.8473)	Low ⊕⊕◯◯	0.1396 (0.0096 ~ 2.0236)	Not estimate	0.2342 (0.0793 ~ 0.6917)	Very low* ⊕◯◯◯
Preop-IM + Postop-IM : Preop EN + Postop-SEN	0.9722 (0.2361 ~ 4.0033)	Moderate ⊕⊕⊕◯		Not estimate	0.9722 (0.2361 ~ 4.0033)	Low* ⊕⊕◯◯
Preop-IM + Postop-IM : Postop-SEN			0.0872 (0.0213 ~ 0.3580)	Moderate ⊕⊕⊕◯	0.0872 (0.0213 ~ 0.3580)	Low* ⊕⊕◯◯
Preop EN + Postop-SEN : Postop-SEN			0.0897 (0.0122 ~ 0.6624)	Not estimate	0.0897 (0.0122 ~ 0.6624)	Not estimate

*, down rating due to imprecision

Supplementary Table S3–3

Comparisons (study)	Direct		Indirect		Network meta-analysis	
	95% CI	Rating of direct evidence	95% CI	Rating of indirect evidence	95% CI	Certainty of evidence
postop EIM : postop TPN	0.9293 (0.3169 ~ 2.7256)	Moderate ⊕⊕⊕◯	4.3737 (0.2376 ~ 80.5161)	Moderate ⊕⊕⊕◯	1.1193 (0.4079 ~ 3.0709)	Low* ⊕⊕◯◯
postop EIM : preop EIM supplementation			1.6163 (0.3361 ~ 7.7720)	Low ⊕⊕◯◯	1.6163 (0.3361 ~ 7.7720)	Very low* ⊕◯◯◯
postop EIM : preop EIM supplementation + postop EIM	9.3333 (0.9410 ~ 92.5742)	Moderate ⊕⊕⊕◯	1.2337 (0.1230 ~ 12.3740)	Moderate ⊕⊕⊕◯	3.4100 (0.6706 ~ 17.3405)	Low* ⊕⊕◯◯
postop EIM : preop EN supplementation + SEN			4.3400 (0.4086 ~ 46.0975)	Moderate ⊕⊕⊕◯	4.3400 (0.4086 ~ 46.0975)	Low* ⊕⊕◯◯
postop EIM : SEN	0.8410 (0.2396 ~ 2.9522)	Moderate ⊕⊕⊕◯	1.0370 (0.1571 ~ 6.8459)	Moderate ⊕⊕⊕◯	0.8968 (0.3153 ~ 2.5512)	Low* ⊕⊕◯◯
postop TPN : preop EIM supplementation			1.4441 (0.3432 ~ 6.0756)	Low ⊕⊕◯◯	1.4441 (0.3432 ~ 6.0756)	Very low* ⊕◯◯◯
postop TPN : preop EIM supplementation + postop EIM	2.6667 (0.2827 ~ 25.1502)	Moderate ⊕⊕⊕◯	3.4271 (0.4158 ~ 28.2469)	Moderate ⊕⊕⊕◯	3.0466 (0.6552 ~ 14.1674)	Low* ⊕⊕◯◯
postop TPN : preop EN supplementation + SEN			3.8775 (0.3879 ~ 38.7624)	Moderate ⊕⊕⊕◯	3.8775 (0.3879 ~ 38.7624)	Low* ⊕⊕◯◯
postop TPN : SEN	0.8250 (0.3816 ~ 1.7838)	Moderate ⊕⊕⊕◯	0.5312 (0.0295 ~ 9.5647)	Moderate ⊕⊕⊕◯	0.8013 (0.3804 ~ 1.6878)	Low* ⊕⊕◯◯
preop EIM supplementation : preop EIM supplementation + postop EIM	1.7101 (0.4047 ~ 7.2264)	Low ⊕⊕◯◯	4.2851 (0.3036 ~ 60.4726)	Not estimate	2.1098 (0.5950 ~ 7.4808)	Very low* ⊕◯◯◯
preop EIM supplementation : preop EN supplementation + SEN			2.6851 (0.3188 ~ 22.6139)	Low ⊕⊕◯◯	2.6851 (0.3188 ~ 22.6139)	Very low* ⊕◯◯◯
preop EIM supplementation : SEN	0.7059 (0.1509 ~ 3.3022)	Low ⊕⊕◯◯	0.2817 (0.0212 ~ 3.7516)	Not estimate	0.5549 (0.1474 ~ 2.0883)	Very low* ⊕◯◯◯
preop EIM supplementation + postop EIM : preop EN supplementation + SEN	1.2727 (0.2292 ~ 7.0661)	Moderate ⊕⊕⊕◯		Not estimate	1.2727 (0.2292 ~ 7.0661)	Low* ⊕⊕◯◯
preop EIM supplementation + postop EIM : SEN			0.2630 (0.0583 ~ 1.1866)	Moderate ⊕⊕⊕◯	0.2630 (0.0583 ~ 1.1866)	Low* ⊕⊕◯◯
preop EN supplementation + SEN : SEN			0.2066 (0.0211 ~ 2.0247)	Not estimate	0.2066 (0.0211 ~ 2.0247)	Not estimate
*, down rating due to imprecision						

Supplementary Table S3–4

Comparisons (study)	Direct		Indirect		Network meta-analysis	
	95% CI	Rating of direct evidence	95% CI	Rating of indirect evidence	95% CI	Certainty of evidence
postop EIM : postop TPN	2.2500 (0.3760 ~ 13.4654)	Moderate ⊕⊕⊕◯	8.1535 (0.0493 ~ 1347.9343)	Moderate ⊕⊕⊕◯	2.5900 (0.4786 ~ 14.0165)	Low* ⊕⊕◯◯
postop EIM : preop EIM supplementation			1.8866 (0.2961 ~ 12.0185)	Low ⊕⊕◯◯	1.8866 (0.2961 ~ 12.0185)	Very low* ⊕◯◯◯
postop EIM : preop EIM supplementation + postop EIM	2.2500 (0.3760 ~ 13.4654)	Moderate ⊕⊕⊕◯	2.0520 (0.0360 ~ 116.8737)	Moderate ⊕⊕⊕◯	4.8583 (0.8088 ~ 29.1821)	Low* ⊕⊕◯◯
postop EIM : SEN			1.0424 (0.1798 ~ 6.0435)	Moderate ⊕⊕⊕◯	1.0424 (0.1798 ~ 6.0435)	Low* ⊕⊕◯◯
postop TPN : preop EIM supplementation			0.7284 (0.2107 ~ 2.5184)	Low ⊕⊕◯◯	0.7284 (0.2107 ~ 2.5184)	Very low* ⊕◯◯◯
postop TPN : preop EIM supplementation + postop EIM	2.6667 (0.3608 ~ 19.7116)	Moderate ⊕⊕⊕◯	1.4008 (0.2264 ~ 8.6672)	Moderate ⊕⊕⊕◯	1.8758 (0.4877 ~ 7.2156)	Low* ⊕⊕◯◯
postop TPN : SEN	0.3859 (0.1935 ~ 0.7699)	Moderate ⊕⊕⊕◯	0.7347 (0.0537 ~ 10.0562)	Not estimate	0.4025 (0.2064 ~ 0.7847)	Low* ⊕⊕◯◯
preop EIM supplementation : preop EIM supplementation + postop EIM	2.3333 (0.8090 ~ 6.7296)	Low ⊕⊕◯◯	4.4418 (0.3682 ~ 53.5829)	Not estimate	2.5752 (0.9716 ~ 6.8253)	Very low* ⊕◯◯◯
preop EIM supplementation : SEN	0.6429 (0.1730 ~ 2.3884)	Low ⊕⊕◯◯	0.3377 (0.0317 ~ 3.5999)	Not estimate	0.5525 (0.1753 ~ 1.7411)	Very low* ⊕◯◯◯
preop EIM supplementation + postop EIM : SEN			0.2146 (0.0574 ~ 0.8023)	Low ⊕⊕◯◯	0.2146 (0.0574 ~ 0.8023)	Very low* ⊕◯◯◯

*, down rating due to imprecision

Supplementary Table S3–5

Comparisons (study)	Direct		Indirect		Network meta-analysis	
	95% CI	Rating of direct evidence	95% CI	Rating of indirect evidence	95% CI	Certainty of evidence
Postop-IM : Postop-TPN	0.7365 (0.2721 ~ 1.9936)	Moderate ⊕⊕⊕◯	1.8492 (0.1912 ~ 17.8826)	Low ⊕⊕◯◯	0.8546 (0.3434 ~ 2.1269)	Low* ⊕⊕◯◯
Postop-IM : Preop-IM			0.4194 (0.0690 ~ 2.5489)	Low ⊕⊕◯◯	0.4194 (0.0690 ~ 2.5489)	Very low* ⊕◯◯◯
Postop-IM : Preop-IM + Postop-IM			0.9034 (0.0717 ~ 11.3889)	Not estimate	0.9034 (0.0717 ~ 11.3889)	Not estimate
Postop-IM : Post-SEN	0.9030 (0.3276 ~ 2.4890)	Moderate ⊕⊕⊕◯	0.3734 (0.0435 ~ 3.2044)	Low ⊕⊕◯◯	0.7689 (0.3074 ~ 1.9238)	Low* ⊕⊕◯◯
Postop-TPN : Preop-IM			0.4908 (0.0961 ~ 2.5069)	Low ⊕⊕◯◯	0.4908 (0.0961 ~ 2.5069)	Very low* ⊕◯◯◯
Postop-TPN : Preop-IM + Postop-IM			1.0571 (0.0946 ~ 11.8123)	Not estimate	1.0571 (0.0946 ~ 11.8123)	Not estimate
Postop-TPN : Post-SEN	0.8998 (0.5492 ~ 1.4744)	Low ⊕⊕◯◯		Moderate ⊕⊕⊕◯	0.8998 (0.5492 ~ 1.4744)	Low* ⊕⊕◯◯
Preop-IM : Preop-IM + Postop-IM	2.1538 (0.3635 ~ 12.7635)	Low ⊕⊕◯◯		Not estimate	2.1538 (0.3635 ~ 12.7635)	Very low* ⊕◯◯◯
Preop-IM : Post-SEN	1.8333 (0.3875 ~ 8.6738)	Low ⊕⊕◯◯		Not estimate	1.8333 (0.3875 ~ 8.6738)	Very low* ⊕◯◯◯
Preop-IM + Postop-IM : Post-SEN			0.8512 (0.0802 ~ 9.0377)	Low ⊕⊕◯◯	0.8512 (0.0802 ~ 9.0377)	Very low* ⊕◯◯◯

*, down rating due to imprecision

Supplementary Table S3–6

Comparisons (study)	Direct		Indirect		Network meta-analysis	
	95% CI	Rating of direct evidence	95% CI	Rating of indirect evidence	95% CI	Certainty of evidence
postop EIM : postop TPN	0.5231 (0.1067 ~ 2.5645)	Moderate ⊕⊕⊕◯	76.5086 (0.0966 ~ 60614.9101)	Low ⊕⊕◯◯	0.6836 (0.1456 ~ 3.2095)	Low* ⊕⊕◯◯
postop EIM : preop EIM supplementation			2.8903 (0.1975 ~ 42.2950)	Low ⊕⊕◯◯	2.8903 (0.1975 ~ 42.2950)	Very low* ⊕◯◯◯
postop EIM : preop EIM supplementation + postop EIM	1.0000 (0.0180 ~ 55.5193)	Moderate ⊕⊕⊕◯	2.2035 (0.0261 ~ 185.7045)	Moderate ⊕⊕⊕◯	1.4278 (0.0727 ~ 28.0225)	Low* ⊕⊕◯◯
postop EIM : SEN	2.0870 (0.1850 ~ 23.5415)	Moderate ⊕⊕⊕◯	0.0862 (0.0063 ~ 1.1872)	Low ⊕⊕◯◯	0.4811 (0.0811 ~ 2.8523)	Low* ⊕⊕◯◯
postop TPN : preop EIM supplementation			4.2281 (0.3903 ~ 45.8012)	Low ⊕⊕◯◯	4.2281 (0.3903 ~ 45.8012)	Very low* ⊕◯◯◯
postop TPN : preop EIM supplementation + postop EIM	1.0000 (0.0180 ~ 55.5193)	Moderate ⊕⊕⊕◯	4.4687 (0.0754 ~ 264.8129)	Moderate ⊕⊕⊕◯	2.0886 (0.1192 ~ 36.5808)	Low* ⊕⊕◯◯
postop TPN : SEN	0.7493 (0.2312 ~ 2.4283)	Low ⊕⊕◯◯	0.1290 (0.0003 ~ 58.4599)	Moderate ⊕⊕⊕◯	0.7037 (0.2218 ~ 2.2330)	Low* ⊕⊕◯◯
preop EIM supplementation : preop EIM supplementation + postop EIM	1.0000 (0.0192 ~ 52.0632)	Low ⊕⊕◯◯	0.2048 (0.0025 ~ 16.9496)	Not estimate	0.4940 (0.0260 ~ 9.3914)	Very low* ⊕◯◯◯
preop EIM supplementation : SEN	0.1289 (0.0119 ~ 1.3916)	Low ⊕⊕◯◯	0.6294 (0.0028 ~ 143.2770)	Not estimate	0.1664 (0.0188 ~ 1.4709)	Very low* ⊕◯◯◯
preop EIM supplementation + postop EIM : SEN			0.3369 (0.0186 ~ 6.0984)	Low ⊕⊕◯◯	0.3369 (0.0186 ~ 6.0984)	Very low* ⊕◯◯◯

*, down rating due to imprecision

Supplementary Table S3–7

Comparisons (study)	Direct		Indirect		Network meta-analysis	
	95% CI	Rating of direct evidence	95% CI	Rating of indirect evidence	95% CI	Certainty of evidence
postop EIM : postop TPN	-3.7000 (-5.6727 ~ -1.7273)	Moderate ⊕⊕⊕◯	-2.9151 (-5.9556 ~ 0.1254)	Low ⊕⊕◯◯	-3.4675 (-5.1224 ~ -1.8126)	Low* ⊕⊕◯◯
postop EIM : preop EIM supplementation			0.9032 (-3.8053 ~ 5.6118)	Moderate ⊕⊕⊕◯	0.9032 (-3.8053 ~ 5.6118)	Low* ⊕⊕◯◯
postop EIM : SEN	-1.9000 (-3.7804 ~ -0.0196)	Moderate ⊕⊕⊕◯	-2.7427 (-6.1496 ~ 0.6642)	Low ⊕⊕◯◯	-2.0968 (-3.7430 ~ -0.4505)	Low* ⊕⊕◯◯
postop TPN : preop EIM supplementation			4.3707 (-0.0793 ~ 8.8207)	Low ⊕⊕◯◯	4.3707 (-0.0793 ~ 8.8207)	Very low* ⊕◯◯◯
postop TPN : SEN	1.3707 (0.7857 ~ 1.9557)	Low ⊕⊕◯◯		Moderate ⊕⊕⊕◯	1.3707 (0.7857 ~ 1.9557)	Low* ⊕⊕◯◯
preop EIM supplementation : SEN	-3.0000 (-7.4114 ~ 1.4114)	Moderate ⊕⊕⊕◯		Not estimate	-3.0000 (-7.4114 ~ 1.4114)	Low* ⊕⊕◯◯

*, down rating due to imprecision
